# Define cancer-associated fibroblasts (CAFs) in the tumor microenvironment: new opportunities in cancer immunotherapy and advances in clinical trials

**DOI:** 10.1186/s12943-023-01860-5

**Published:** 2023-10-02

**Authors:** Hao Zhang, Xinghai Yue, Zhe Chen, Chao Liu, Wantao Wu, Nan Zhang, Zaoqu Liu, Liping Yang, Qing Jiang, Quan Cheng, Peng Luo, Guodong Liu

**Affiliations:** 1 Department of Neurosurgery, The Second Affiliated Hospital, Chongqing Medical University, Chongqing, China; 2 Department of Urology, The Second Affiliated Hospital, Chongqing Medical University, Chongqing, China; 3 Department of Neurosurgery, Central Hospital of Zhuzhou, Zhuzhou, China; 4 Department of Oncology, Xiangya Hospital, Central South University, Changsha, China; 5 College of Life Science and Technology, Huazhong University of Science and Technology, Wuhan, China; 6 Department of Interventional Radiology, The First Affiliated Hospital of Zhengzhou University, Zhengzhou, China; 7 Department of Laboratory Medicine, The Second Affiliated Hospital, Chongqing Medical University, Chongqing, China; 8 Department of Neurosurgery, Xiangya Hospital, Central South University, Changsha, China; 9 National Clinical Research Center for Geriatric Disorders, Xiangya Hospital, Central South University, Changsha, China; 10 Department of Oncology, Zhujiang Hospital, Southern Medical University, Guangzhou, China

**Keywords:** CAF, Clinical trial, Microenvironment, Immunotherapy, Target

## Abstract

Despite centuries since the discovery and study of cancer, cancer is still a lethal and intractable health issue worldwide. Cancer-associated fibroblasts (CAFs) have gained much attention as a pivotal component of the tumor microenvironment. The versatility and sophisticated mechanisms of CAFs in facilitating cancer progression have been elucidated extensively, including promoting cancer angiogenesis and metastasis, inducing drug resistance, reshaping the extracellular matrix, and developing an immunosuppressive microenvironment. Owing to their robust tumor-promoting function, CAFs are considered a promising target for oncotherapy. However, CAFs are a highly heterogeneous group of cells. Some subpopulations exert an inhibitory role in tumor growth, which implies that CAF-targeting approaches must be more precise and individualized. This review comprehensively summarize the origin, phenotypical, and functional heterogeneity of CAFs. More importantly, we underscore advances in strategies and clinical trials to target CAF in various cancers, and we also summarize progressions of CAF in cancer immunotherapy.

## Introduction

The tumor microenvironment (TME) has been studied in depth with the progression of research on solid tumors. TME refers to the surrounding microenvironment tumor cells reside and develop, including surrounding blood vessels, the extracellular matrix (ECM), multiple signaling molecules, and non-neoplastic cells like immune cells, fibroblasts, lipocytes, etc. [1, 2]. Among all those various TME components, cancer-associated fibroblasts (CAFs) have been noted to exhibit a higher correlation with tumor development and have become a hot spot for oncology research [3].

CAFs are widely known for their significant heterogeneity, which is reflected explicitly in the substantial subpopulation of CAFs [4], as well as the juxtaposition of tumor-promoting and tumor-restraining [5]. As a substantial component of tumor stroma, CAFs perform an essential function in tumor progression and metastasis [6], including ECM depositing and remodeling [7], having crosstalk with immune cells [8], promoting cancer cell proliferation, angiogenesis, and drug resistance [9–11]. Simultaneously, some research indicates that CAFs could exert tumor-restraining functions in particular cancer types [12, 13].

Studies of interaction with TME identified numerous mechanisms, thus presenting multiple potential targets for oncotherapy. Nevertheless, various clinical trials of treatment strategies targeting CAFs have failed and, in some cases, even culminated in accelerating cancer progression and resulting in shortened patient survival [5]. The reasons for this are that the role of CAFs in tumorigenesis and development has not been fully elucidated, and that the function of CAFs is context-dependent and has significant plasticity. Therefore, more research is urgently needed to investigate the potential of CAFs as therapeutic targets for oncotherapy.

This review will initially summarize the background knowledge of CAFs, especially their heterogeneity and the pro-tumor functions of CAFs, including angiogenesis, metastasis, extracellular matrix remodeling, immunosuppression, etc. We will also introduce the current status of research on CAF as a potential tumor therapeutic target. Finally, we will present the latest advances in oncology therapeutic research and clinical trials for CAF in several cancer types.

## Background knowledge of CAFs

### Overview of TME

With the deepening of cancer research, increasing evidence continuously proves that TME is closely pertaining to nearly all stages of cancer, and the existing research model has gradually changed from tumor-centric to TME-centric. TME is typically defined as a multicellular niche characterized by a hypoxic, acidic environment. The main cellular components include immune cells such as T and B lymphocytes, macrophages, dendritic cells (DC), natural killer (NK) cells, and myeloid-derived suppressor cells (MDSC); stromal cells like CAFs, pericytes, and mesenchymal stromal cells; other non-cellular components like ECM, blood vessels, lymphoid organs or lymph nodes, nerves, and chemokines. The classification of T cells is complex and will not be described in detail here. T cells in TME mainly include CD4^+^ T cells, CD8^+^ T cells, Tregs, and γδ T cells. CD8^+^ T cells are robust effector cells that release granzyme and perforin-induced apoptosis in tumor cells. CD4^+^ T cells are helper T cells, divided into th1 and th2 types, and their effects are also opposed. Treg is the key to maintaining immune balance in the body and mainly plays an anti-tumor role in TME. γδ T cells are a specialized subset of T cells that express γδTCR and recognize target antigens in an MHC-independent manner. γδT cells also play a dual role, secreting IL-17 to inhibit the anti-tumor immune response and also exerting cytotoxic effects to kill tumor cells [14–18]. B cells are another large class of specific immune cells, majorly involved in humoral immunity. The dual effect of B cells on tumors is manifested by secreting pro-inflammatory factors, activating complement to suppress immunity, and directly killing tumor cells [19]. As for macrophages, they are divided into two subgroups, M1 has antitumor effects, and M2, on the contrary, promotes tumor development by suppressing immunity, promoting angiogenesis and metastasis. T cells, B cells, and antigen-presenting cells are collectively called specific immune cells, of which dendritic cells are a type of antigen presenting cell (APC) that integrates information from TME and transmits it to other immune cells [20]. Mast cells are a type of granulocytes that play an important role in type 1 hypersensitivity and autoimmunity, and they can secrete cytokines in TME that promote angiogenesis, tumor invasion, and kill tumor cells [21]. NK cells are non-specific immune cells that can kill tumor cells in a variety of ways, hence they have a strong anti-cancer ability. But tumor cells can escape by, for example, inhibiting the upregulation of receptors [22]. Moreover, monocytes are precursors of macrophages and dendritic cells, and neutrophils, eosinophils, and basophils are all granular leukocytes, which also have dual functions of anti-tumor and tumor suppression [23–26]. Mesenchymal stromal cells are derived from the mesoderm in early development, with self-replication ability and multidirectional differentiation potential. It secretes TGF-β and other chemical factors in TME to promote tumor progression and also has tumor cytotoxicity to inhibit tumor growth. Myeloid-derived suppressor cells, composed of immature monocytes and neutrophils, can inhibit the function of a wide range of immune cells and are therefore associated with poor clinical outcomes [27, 28]. Pericytes are adjacent to endothelial cells, and they have been reported to be associated with TME immunosuppressive states and epithelial-to-mesenchymal transition (EMT). Lastly, adipocytes are closely related to cancer cells. They release free fatty acids, hormones, cytokines, adikines, and growth factors that affect cancer cells and host cells in TME [29, 30].

These biological constituents do not function independently but interact to influence tumor progression by secreting various chemical factors, chemokines, exosomes, etc. Briefly, the cellular composition and functional status of TME varies depending on a range of conditions such as the site of tumorigenesis, the inherent characteristics of cancer cells, tumor stage, and patient features. Alterations in TME are inseparable from crosstalk between tumor cells and cellular components within TME. As one of the most abundant cell types in TME, CAF is the center of cross-communication among various cells in the tumor stroma. Analogous to most of the abovementioned cells, the fact that CAFs display both pro-tumor and anti-tumor functions within TME is not unexpected. The tumor-promoting function of CAF is multifaceted, such as participating in the reconstruction of ECM and the formation of  the immunosuppressive microenvironment, but its specific subtype has also been observed to have tumor suppressive function, which will be described in more detail later [31–33]. The functional differences between CAF and other cells within TME are roughly summarized in Table 1 [14–17, 19–30, 34–52].

**Table 1:** Cellular components and corresponding functions within TME

Cell type		Biomarker(s)	Functions in the TME	References
T cells	CD4^+^ T cells	CD4	(1) Inducing CD8^+^ T cell responses; (2) Secreting interferon γ (IFNγ) and tumor necrosis factor (TNF); (3) Releasing anti-inflammatory factors	[14]
	CD8^+^ T cells	CD8	(1) Specific recognition of antigenic peptides-MHC class I molecular complexes; (2) Secreting IFN-γ and the protease granzyme B; (3) Killing cancer cells via perforin-mediated apoptosis and FASL-FAS-mediated cell death; (4) Noncytolytic subsets have also been identified	[15, 34]
	Tregs	CD25, Foxp3	(1) Secreting inhibitory cytokines; (2) Killing effector cells by granzymes and perforin; (3) Affecting effector cell function; (4) Reinforcing immunosuppression	[16]
	γδ T cells	CD3, γδ	(1) Recognizing antigen in an MHC-independent way; (2) Directly recognizing cancer cells through TCR and/or natural killer cell receptors (NKRs); (3) Indirectly displaying antitumor function via influencing downstream immune responses; (4) Producing IL-17 to enhance tumorigenicity	[17]
B cells		CD19	(1) Following antigen presentation; (2) Activating T cells; (3) Recognition of tumor antigens by BCRs and direct killing of tumor cells; (4) Generation of pro-inflammatory factors like IL-1β (5) Inhibiting anti-cancer immunity through promotion of immune tolerance and direct suppression of T cells	[19]
Macrophages	M1	CD86, CD64, MARCO	(1) Direct cytotoxicity; (2) Tumor cell elimination through ADCC	[20, 35]
	M2	CD206, CD163, ARG1	(1) Immunosuppression; (2) Releasing chemokines such as VEGFA, PDGF, MMPs, HIF, IL-10, COX2, and adrenomedullin to facilitate angiogenesis and lymphangiogenesis; (3) Promoting metastasis; (4) supporting drug resistance	[20, 36]
Dendritic cells (DCs)		CD11c, HLA-DR	(1) Presenting antigen; (2) Inducing and maintaining CD8^+^ T cell responses	[37, 38]
Mast cells		CD32, CD33, CD117	(1) Promoting angiogenesis via secreting factors like IL-8, NGF, TGF-β VEGF-A, and VEGF-B; (2) Releasing MMPs to support tumor invasion; (3) Eliminating tumor cells through secreting IL-1, IL-4, IL-6, and TNF-α; (4) Enhancing tumor expansion via secreting FGF-2, NGF, PDGF, VEGF, IL-8, and IL-10	[21, 39]
NK cells		CD56, NKp46, CD94	(1) Tumor cytotoxic activity; (2) Killing cancer cells via the ‘missing-self’ mechanism; (3) Secreting cytokines like IFN-γ and TNF-α	[22, 40]
Monocytes		CD14	(1) Differentiating into TAMs, tumor-associated DCs (TADCs), and MDSCs; (2) Tumor cytotoxicity; (3) Activating APCs; (4) Potentiating angiogenesis and reshaping ECM (5) Immunosuppression	[23, 41]
Neutrophils		CD11b, CD16	(1) Directly killing cancer cells and trogoptosis; (2) Altering TME through improving T lymphocyte response and influencing macrophages; (3) releasing ROS and PGE_2_; (4) Immunosuppression; (5) Promoting cancer angiogenesis	[24]
Eosinophils		CD125, Siglec-8^+^	(1) Tumor cell toxicity; (2) Vessel normalization; (3) Secreting various soluble factors such as IL-10, IL-12 and cytotoxic proteins, such as MBP and ECP; (4) favoring tumor progression via remodeling ECM, inducing macrophage polarization, and suppressing immune response	[25]
Basophils		CD22, CD123	(1) Releasing proangiogenic factors like VEGF-A, VEGF-B, ANGPT1, and HGF; (2) Secreting granzyme B, TNF-α, and histamine	[26, 42–44]
Mesenchymal stromal cells (MSCs)		CD105, CD73, CD90	(1) Promoting tumor growth and progression via secreting cytokines and chemokines like VEGF, TGF-β1, IL-6 and IL-8; (2) Tumor toxicity and TLR expression; (3) Exosome releasing	[27, 45, 46]
Myeloid-derived suppressor cells (MDSCs)		CD11b, Gr 1, Ly6G, Ly6C	(1) Suppressing T-cell through the high expression of ARG1, iNOS, and ROS and depletion of required amino acids; (2) Inhibiting NK cells, DCs, and B cells; (3) Promoting vascularization and pre-metastatic niche formation	[28, 47, 48]
Cancer-associated fibroblasts (CAFs)		FAP, α-SMA	(1) Promoting tumor growth, angiogenesis, metastasis, and drug resistance; (2) Remodeling ECM; (3) Suppressing immunity; (4) Cancer-restraining function	[49, 50]
Pericytes		PDGFR-β, αSMA, CD146, NG2	(1) Modulating immunosuppressive TME; (2) Forming the pre-metastatic niche; (3) Participating in EMT	[29, 51]
Adipocytes		ASC-1	(1) Secreting pro-inflammatory cytokines, adiponectin, and autotaxin (ATX); (2) Altering cancer cell metabolism	[30, 52]

*Abbreviations*: *ADCC* Antibody-dependent cell-mediated cytotoxicity, *ANGPT1* Angiopoietin 1, *APC* Antigen presenting cell, *ARG1* Arginase 1, *BCR* B cell receptor, *CD* Clusters of differentiation, *COX2* Cyclooxygenase 2, *CXCL8* C–X–C motif chemokine ligand 8, *ECM*, Extracellular matrix, *ECP* Eosinophil cationic protein, *EMT* Epithelial-to-mesenchymal transition, *FAP* Fibroblast activation protein, *FAS* Factor-related apoptosis, *FOXP3* Forkhead box protein P3, *HGF* Hepatocyte growth factor, *HIF* Hypoxia-inducible factor, *iNOS* Inducible-NO synthase, *MBP* Major basic protein, *MMPs* Matrix metalloproteinases, *NGF* Nerve growth factor, *PDGFR-β* Platelet-derived growth factor receptor β, *PGE*_*2*_ Prostaglandin E2, *ROS* Reactive oxygen species, *TCR* T cell receptor, *TLR* Toll-like receptor, *VEGF-A* Vascular endothelial growth factor-A, *VEGF-B* Vascular endothelial growth factor-B, *α-SMA* α-smooth muscle actin

### Biomarkers, origins and regulation of CAFs

#### Biomarkers of CAFs

Generally, fibroblasts are defined as interstitial cells of the mesenchymal lineage. Fibroblasts are cells that produce collagen and contribute to the formation of connective tissues, which help maintain the typical structure of tissues. Quiescent fibroblasts are activated during wound healing and neoplasia. As a result, the currently widely accepted hypothesis is that CAFs are activated by fibroblasts located in or near tumors in the context of tumors, which is also why they are called CAFs [3, 5, 53].

Several different biomarkers are used to define CAFs, including but not limited to α-smooth muscle actin (α-SMA, also known as ACTC2), platelet-derived growth factor receptorα/β (PDGFRα/β), fibroblast-specific protein 1 (FSP-1, also known as S100A4), fibroblast activation protein (FAP) [54–57]. Nevertheless, the specific biomarker that can define all sorts of CAFs has not been found yet. Among these biomarkers, FAP, a type II transmembrane glycoprotein, whose expression was selectively observed in CAFs and pericytes in most human epithelial cancers, was thought to facilitate tumor growth and proliferation [58, 59]. To date, it is extensively considered to be the most promising target of CAF-based oncotherapy [58, 60, 61]. Depletion of FAP-positive fibroblasts caused necrosis of both tumor and stroma cells in a transgenic mouse model of lung cancer [62], which reflected the tumor-promoting function of FAP from another aspect. More information on the progress of the treatment will be provided in detail later.

#### Origins of CAFs

**Fig. 1:**
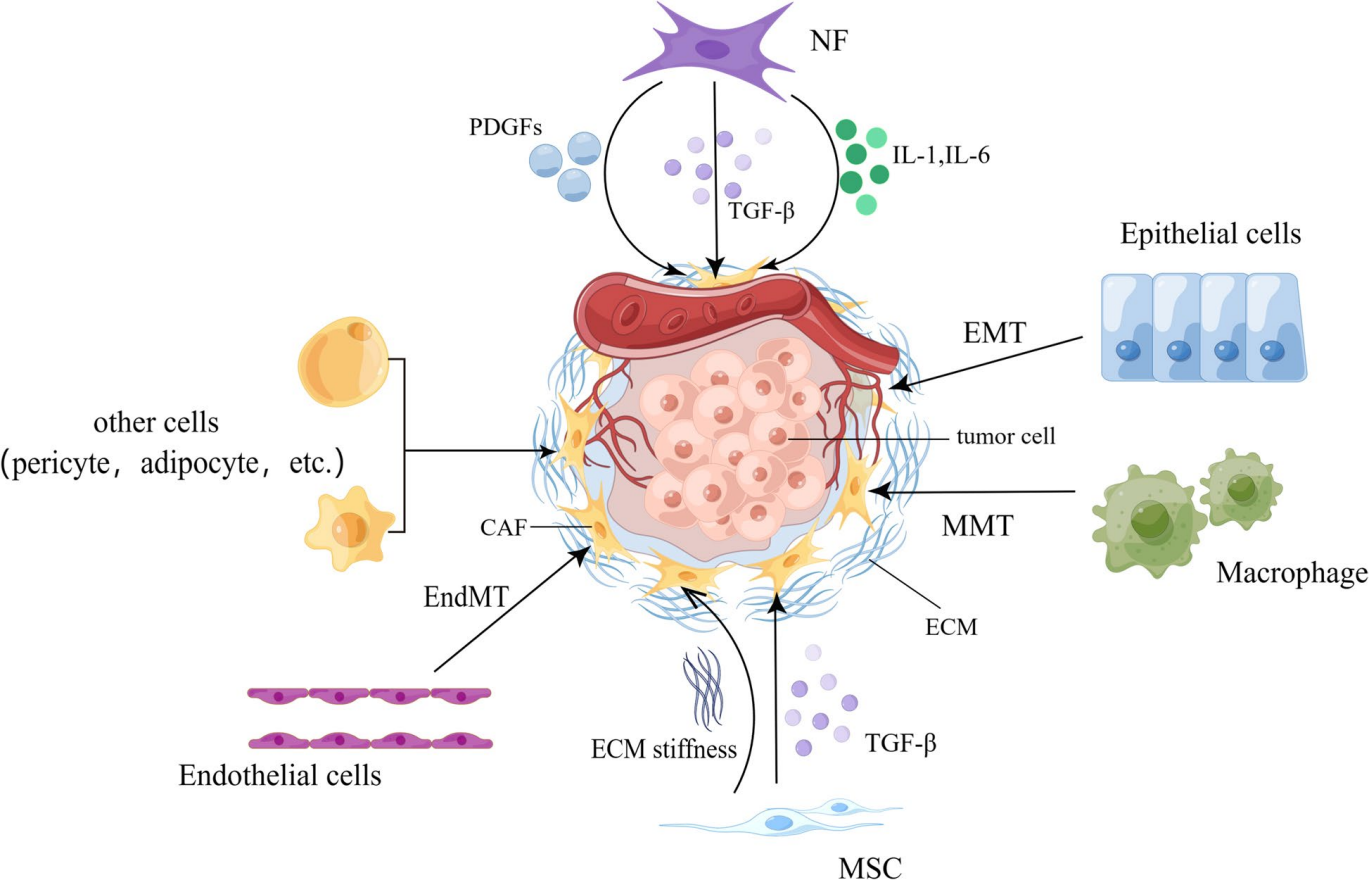
Possible origins of CAF. ECM, extracellular matrix; EMT, epithelial-to-mesenchymal transition; EndMT, endothelial-to-mesenchymal transition; IL, interleukin; MMT, macrophage-to-mesenchymal transition; MSC, mesenchymal stem cells; NF, normal fibroblast; PDGF, platelet-derived growth factor; TGF-β, transforming growth factor beta. By Figdraw

As a result of being devoid of specific biomarkers to define all CAFs, the precise cellular origin of CAFs still needs to be fully elucidated. Lineage tracing studies showed many putative cellular precursors [49, 63]. CAFs can derive from resident fibroblasts [64]. These resident fibroblasts, quiescent pancreatic stellate cells (PSCs), and hepatic stellate cells (HSCs) can acquire a myofibroblast-like phenotype in the liver and pancreas, including α-SMA expression. In that case, these two cells are considered CAFs in pancreatic and liver cancers, respectively [65, 66]. Despite the local cellular sources, CAFs can also originate from bone marrow-derived mesenchymal stem cells (BM-MSCs) [27], which is confirmed by both *in vitro* and *in vivo* tracing studies [67–69]. Epithelial and endothelial cells were reported to adopt a fibroblastic phenotype with the expression of S100A4 through EMT and endothelial-to-mesenchymal transition (EndMT), respectively, making them a possible origin of CAFs [56, 70]. The expression of CAF- and EMT-related markers and proteins has also been highly correlated with the progression of skin malignancies [71]. Moreover, recent studies have demonstrated in non-small cell lung cancer (NSCLC) that CAFs can derive from macrophage via macrophage-myofibroblast transition (MMT), which is also relevant to fibrotic nephropathy [72, 73]. Except for the aforementioned familiar sources, other possible sources include pericytes [74] and adipocytes [75] (Fig. 1).

#### Regulation of CAFs

In particular, researchers have concentrated on dissecting the modification process of NF (normal fibroblast)-CAF transition for a long time. It is believed that the genome of CAF is relatively stable, and transcriptional regulation plays an instrumental role in reprogramming. Lee, K.-W. et al*.* demonstrated the existence of a master transcription factor (mTF) PRRX1 in vivo and in vitro, which closely pertained to fibroblast acquisition of the CAF phenotype. Transcription factor SOX2 was revealed to participate in this process as well [76–78]. DNA methylation and histone methylation/acetylation are two major alterations within epigenetic modifications that affect the transcriptional factors. In cancer cells, the presence of global DNA hypomethylation and local DNA hypermethylation were both observed, and similar patterns were found in CAFs by numerous research. Recent investigations have shown that CAF DNA methylation depends on the kind of cancer, with some CAFs having abnormal, not just reduced, methylation, even if the trend of global DNA hypomethylation persists in CAFs from many malignancies. Moreover, recent research has emphasized the significance of histone methylation for CAF function. For instance, during enhancer reprogramming, the histone acetylation and methylation mark histone H3 lysine 27 acetylation (H3K27ac) and histone H3 lysine 4 mono-methylation (H3K4me1) was found, accompanied with CAF activation [79]. The loss of S-adenosyl methionine-mediated histone hypomethylation caused nicotinamide N-methyltransferase (NNMT) production in CAFs to enhance cytokine secretion and ECM deposition in ovarian cancer. Additionally, CAFs contain several mediators of epigenetic control. TGF-β, LIF, JAK1/STAT3, IL-1, IL-1, TNF-α, IL-6, and HIF-1 are well-known soluble factors that activate CAF. The surrounding TME drives the change from NFs to CAFs during CAF maturation. The miRNAs' ability to contribute to and adapt to the surrounding milieu has led to their involvement in this transient process. Examples of these miRNAs are miR-149, miR-27a, miR-29a-3p, miR-30c-5p, and miR-200 s/miR-221 [78, 80].

### Heterogeneity of CAFs

#### Cellular phenotype heterogeneity of CAFs

Studies of human cancers and mouse models using immunostaining, in situ hybridization, flow cytometry, fluorescence-activated cell sorting, and mRNA microarrays validated the existence of distinct CAF subsets. More recently, thanks to the emergence and application of single-cell RNA sequencing (scRNA-seq), cellular heterogeneity has been detected, improving the resolution of gene expression studies, which enables a deeper understanding of CAF subsets in different tumor types [4]. By analyzing a wide range of biomarkers selectively expressed on the surface of CAFs in specific TMEs, numerous CAF phenotypes have been defined in different cancers, reflecting the significant phenotypical heterogeneity of the CAFs [81–94].

In pancreatic ductal adenocarcinoma (PDAC), Öhlund and colleagues distinguished two distinct CAF subpopulations, inflammatory CAFs (iCAFs) and myofibroblastic CAFs (myCAFs). iCAFs exhibited low expression of αSMA and high expression of cytokines such as IL6, IL11, and PDGFRα [81]. Moreover, iCAFs were reported to be induced by circCUL2/ microRNA (miR) -203a-5p/NF-κB/IL6 axis from NFs [95]. In contrast, FAP^+^ myCAFs had a selectively high expression of αSMA and lacked expression of inflammatory cytokines. The spatial distribution discrepancy of these two CAF subsets was observed via immunostaining of tumor organoids. Specifically, myCAFs were located near neoplastic cells, whereas iCAFs were more distant [81]. Despite PDAC, these two CAF subpopulations were also defined in other cancer contexts [94, 96]. In another study, Elyada et al. identified ‘antigen-presenting CAFs (apCAFs)’, which expresses Major Histocompatibility Class (MHC) II and CD74 but no classic costimulatory molecules (CD80, CD86, CD40), in KPC tumors by using scRNA-seq and immunohistochemical analysis. Researchers revealed that apCAFs were able to activate CD4^+^ T cells in an antigen-specific fashion, confirming their putative immune-modulatory capacity [92]. Another scRNA-seq of fibroblasts from different stages of KIC tumors found that mesothelial cells in the normal pancreas had a similar genetic profile to apCAFs, suggesting that apCAFs may originate from mesothelial cells [97].

Concomitant analyses of six biomarkers, including FAP, CD29 (integrin-β1), α-SMA, FSP1, PDGFRβ, and caveolin, characterized four CAF subsets (from CAF-S1 to CAF-S4) with distinct properties in ovarian and breast cancers (BC) [94, 98]. Kieffer et al. further identified eight different CAF-S1 clusters (from cluster 0 to cluster 7) in BC by using scRNA-seq. Two of these CAF -S1 clusters, namely ECM-myCAF and TGFβ-myCAF, were found to play an imperative role in forming an immunosuppressive environment and resisting immunotherapy. ECM-myCAF was demonstrated to stimulate the expression of both PD-1 and CTLA-4 protein at the surface of CD4^+^ CD25^+^ T lymphocytes, and PD-1^+^ CTLA4^+^ Tregs can reciprocally alter the proportion of TGFβ-myCAF through converting ECM-myCAF into TGFβ-myCAF [93]. In 2018, researchers observed that CAFs in the MMTV-PyMT mouse model of BC can be classified into four distinct categories: vascular CAFs (vCAFs), matrix CAFs (mCAFs), cycling CAFs (cCAFs), and developmental CAFs (dCAFs). Among them, vCAF was derived from the perivascular area and mCAFs originated from resident fibroblasts, and these subsets were also of different clinical significance [83]. Ds, F. et al*.* identified six transcriptionally distinct clusters of CAFs in endogenous mouse breast tumors. They further signified three major clusters using spatial transcriptomics, which were mechanoresponsive (MR) CAF, steady state-like (SSL) CAF, and immunomodulatory (IM) CAF, and these subpopulations were found conservative across multiple solid tumor tissues and species [86]. More recently, single-cell transcriptomics revealed that CAF in BC originates from CD26^+^ and CD26^−^ NF populations, and then they differentiated into specific functional subpopulations [99].

The application of scRNA-seq in human gastric cancer (GC) has identified a prior undetected subset of CAF, characterized by high expression of Periostin (POSTN), which encodes a protein that functions as an adhesion-modulating factor in the ECM component. This CAF subpopulation is highly expressed in genes involved in ECM remodeling and is therefore defined as extracellular matrix CAFs (eCAFs). Furthermore, tumor-derived eCAFs, as important components in TME to promote metastasis, are inseparable from the increase in gene expression associated with tumor invasion. Simultaneously, high POSTN expression is associated with GC patients’ unsatisfactory overall survival (OS), demonstrating its potential value in predicting prognosis [96]. Lambrechts and colleagues defined seven subsets of fibroblasts by scRNA-seq analysis of stromal cells derived from excised NSCLC tumor tissue and non-tumor lung tissue. They identified five types of fibroblasts in cancerous tissue and detected marker genes for each subpopulation [88]. Lastly, a study conducted by Galbo, P. M. and colleagues identified six CAF subtypes that are generally observed in melanoma, head and neck squamous cell carcinoma, and lung cancer. Specific subpopulations including pan-myCAF, pan-dCAF, pan-iCAF, pan-pCAF, and pan-iCAF-2 were found pertaining to immunotherapy resistance [91]. More information about CAF phenotypic heterogeneity is summarized in Tables 2.

**Table 2:** CAF Phenotypes across various cancers

Tumor type	Sample type	CAF Phenotype		Biomarker(s)	Ref
Pancreatic ductal adenocarcinoma	Patient, KPC mouse	myCAF		α-SMA^High^, FAP	[81, 92]
		iCAF		IL-6, IL11, α-SMA^Low^, PDGFRα^High^	
		apCAF		MHCII, CD74	
Breast cancer and ovarian cancer	Patient	CAF-S1		CD29^Med^ FAP^High^ FSP1^Low−High^ α-SMA^High^ PDGFRb^Med−High^ CAV1^Low^	[93, 94]
		CAF-S2		CD29^Low^ FAP^Neg^ FSP1^Neg−Low^ α-SMA^Neg^ PDGFRb^Neg^ CAV1^Neg^	
		CAF-S3		CD29^Med^ FAP^Neg^ FSP1^Med−High^ α-SMA^Neg−Low^ PDGFRb^Med^ CAV1^Neg−Low^	
		CAF-S4		CD29^High^ FAP^Neg^ FSP1^Low−Med^ α-SMA^High^ PDGFRb^Low−Med^ CAV1^Neg−Low^	
Breast cancer	Patient	cluster 0 (ECM-myCAF)		LRRC15, GBJ2	[93]
		cluster 1 (detox-iCAF)		ADH1B, GPX3	
		cluster 2 (IL-iCAF)		RGMA, SCARA5	
		cluster 3 (TGFβ-myCAF)		CST1, TGFb1	
		cluster 4 (wound-myCAF)		SEMA3C, SFRP4	
		cluster 5 (IFNg-i CAF)		CCL19, CCL5	
		cluster 6 (IFNab-myCAF)		IFIT3, IRF7	
		cluster 7 (acto-myCAF)		GGH, PLP2	
Breast cancer	Patient	CAF +		ADAMTS12^High^, AEBP1^High^, COL10A1^High^, COL11A1^High^, EDNRA^High^, EPPK1^High^, WNT7B ^High^, CXCL11^Low^, CXCR6^Low^	[82]
		CAF-		ADAMTS12^Low^, AEBP1^Low^, COL10A1 ^Low^, COL11A1^Low^, EDNRA^Low^, EPPK1^Low^, WNT7B ^Low^ CXCL11 ^High^, CXCR6^High^	
Breast cancer	Murine	vCAF		FAP, S100a4, ACTA2, PDGFRβ	[83]
		mCAF		FAP, S100a4, ACTA2, PDGFRα	
		cCAF		FAP, S100a4, ACTA2, PDGFRβ	
		dCAF		FAP, Sox9, Sox10, PDGFRβ	
Solid tumors	Murine and human	SSL CAF	Cluster 1	Pi16, Dpp4, Dpt, CD34	[86]
			Cluster 3	Pi16, Dpp4	
		MR CAF	Cluster 2	FAK	
			Cluster 4	Lrrc15, Spp1	
			Cluster 5	Thbs 2, FSP 1, Col 6a 1, Cdh 11	
		IM CAF	Cluster 0	IL 1, IFNg, CXCL12	
Gastric cancer	Patient	iCAF		IL6, CXCL12	[96]
		eCAF		MMP14, LOXL2, POSTN	
Colorectal cancer	Human	CAF-A		FAP, MMP2, DCN	[84, 85]
		CAF-B		α-SMA, PDGFA, TAGLN	
Lung cancer	Patient	subtype I		HGF^High^ FGF7^High/Low^ p-SMAD2^Low^	[87]
		subtype II		HGF^Low^ FGF7^High^ p-SMAD2^Low^	
		subtype III		HGF^Low^ FGF7^Low^ p-SMAD2^High^	
Lung cancer	Patient	Cluster1		COL10A1	[88]
		Cluster2		COX4I2	
		Cluster3		–	
		Cluster4		PLA2G2A	
		Cluster5		MMP3	
		Cluster6		FIGF	
		Cluster7		–	
Metastatic lymph nodes	Patient	CAF-S1		FAP^High^ CD29^Med−High^ αSMA^High^ PDPN^High^ PDGFRβ^High^;	[89]
		CAF-S2		FAP^Neg^ CD29^Low^ αSMA^Neg−Low^ PDPN^Low^ PDGFRβ^Low^	
		CAF-S3		FAP^Neg−Low^ CD29^Med^ αSMA^Neg−Low^ PDPN^Low^ PDGFRβ^Low−Med^	
		CAF-S4		FAP^Low−Med^ CD29^High^ αSMA^High^ PDPN^Low^ PDGFRβ^Med^	
Ovarian cancer	Patient	CAF_c1		CCDC80, SFRP2, VCAN, COL8A1	[90]
		CAF_c2		RGS5, NOTCH3, NDUFA4L2	
Melanoma, head and neck squamous cell carcinoma, and lung cancer	Patient	Pan-CAF 1(pan-myCAFs)		ACTA2, MYH11, MCAM, TAGLN	[91]
		Pan-CAF 2(pan-dCAFs)		COL1A1, COL3A1	
		Pan-CAF 3(pan-iCAFs)		CFD, CXCL14, CXCL12	
		Pan-CAF 4(pan-iCAFs-2)		CXCL2	
		Pan-CAF 5(pan-nCAF)		CXCR4, NCAM1	
		Pan-CAF 6(LQ-CAF)		C10orf10	
		Pan-CAF 7(pan-pCAF)		BIRC5, TOP2A	

*Abbreviations*: *ACTA2* Actin alpha 2, *ADAMTS12* ADAM metallopeptidase with thrombospondin type 1 motif 12, *ADH1B* Alcohol dehydrogenase 1, *AEBP1* Adipocyte enhancer-binding protein 1, *BIRC5* Baculoviral IAP repeat containing 5, *CAV* Caveolin, *CCDC80* Coiled-coil domain-containing protein 80, *CCL* CC motif chemokine ligand, *CD* Clusters of differentiation, *Cdh 11* Cadherin 11, *CFD* Complement factor D, *COL1* Collagen type I, *COL10A1* Collagen type X alpha 1 chain, *COX4I2* Cytochrome C oxidase subunit 4I2, *CST1* Cystatin, *CXCL11* C–X–C motif chemokine ligand 11, *CXCR6* C-X-C motif chemokine receptor 6, *C10orf10* Chromosome 10 open reading frame 10, *DCN* Decorin, *Dpp4* Dipeptidyl peptidase 4, *Dpt* Dermatopontin, *EDNRA* Endothelin receptor type A, *EPPK1* Epiplakin 1, *FAK* Focal adhesion kinase, *FGF7* Fibroblast growth factor 7, *FIGF* Vascular endothelial growth factor D, VEGF-D, *FSP 1* Ferroptosis suppressor protein 1, *GBJ2* Gap junction protein beta 2, *GGH* G-glutamyl hydrolase, *GPX3* Glutathione peroxidase 3, *IFIT3* Interferon induced protein with tetratricopeptide repeats 3, *IFNg* Interferon gamma, *IL* Interleukin, *IRF7* Interferon regulatory factor 7, *LRRC 15* Leucine rich repeat containing 15, *Med* Medium, *MCAM* Melanoma cell adhesion molecule, *MHCII* Major histocompatibility complex class II, *MMP* Matrix metalloproteinase, *MYH1* Myosin heavy chain 1, *NCAM1* Neural cell adhesion molecule 1, *NDUFA4L2* NDUFA4 mitochondrial complex associated like 2, *Neg* Negative, *NOTCH3* Neurogenic locus notch homolog protein 3, *PDGFR* Platelet-derived growth factor receptor, *PDPN* Podoplanin, *Pi16* peptidase inhibitor 16, *PLA2G2A* Phospholipase A2 group IIA, *PLP2* Proteolipid protein 2, *RGMA* Repulsive guidance molecule BMP co-receptor, *RGS5* Regulator of G protein signaling 5, *SCARA5* Scavenger receptor class A member 5, *SEMA3C* Semaphorin 3C, *SFRP2* Secreted frizzled-related protein 2, *Sox* SRY-related HMG-box, *Spp1* Secreted phosphoprotein 1, *TAGLN* Transgelin, *Thbs 2* Thrombospondin 2, *TOP2A* DNA topoisomerase II alpha, *VCAN* Versican, *WNT7B* Wnt family member 7B, *α-SMA* Alpha-smooth muscle actin

Currently, defining functional populations of CAFs using cell surface biomarkers is still a challenging task. Because the cell source of CAF is not monolithic, it is almost impossible to identify universal CAF markers across different cancer types. Future studies could combine scRNA-seq and in vivo models to better elucidate the heterogeneity of CAF in the context of cell origin, surface markers, RNA profiles, activation phases, and spatial distribution.

#### Functional heterogeneity of CAFs

**Fig. 2:**
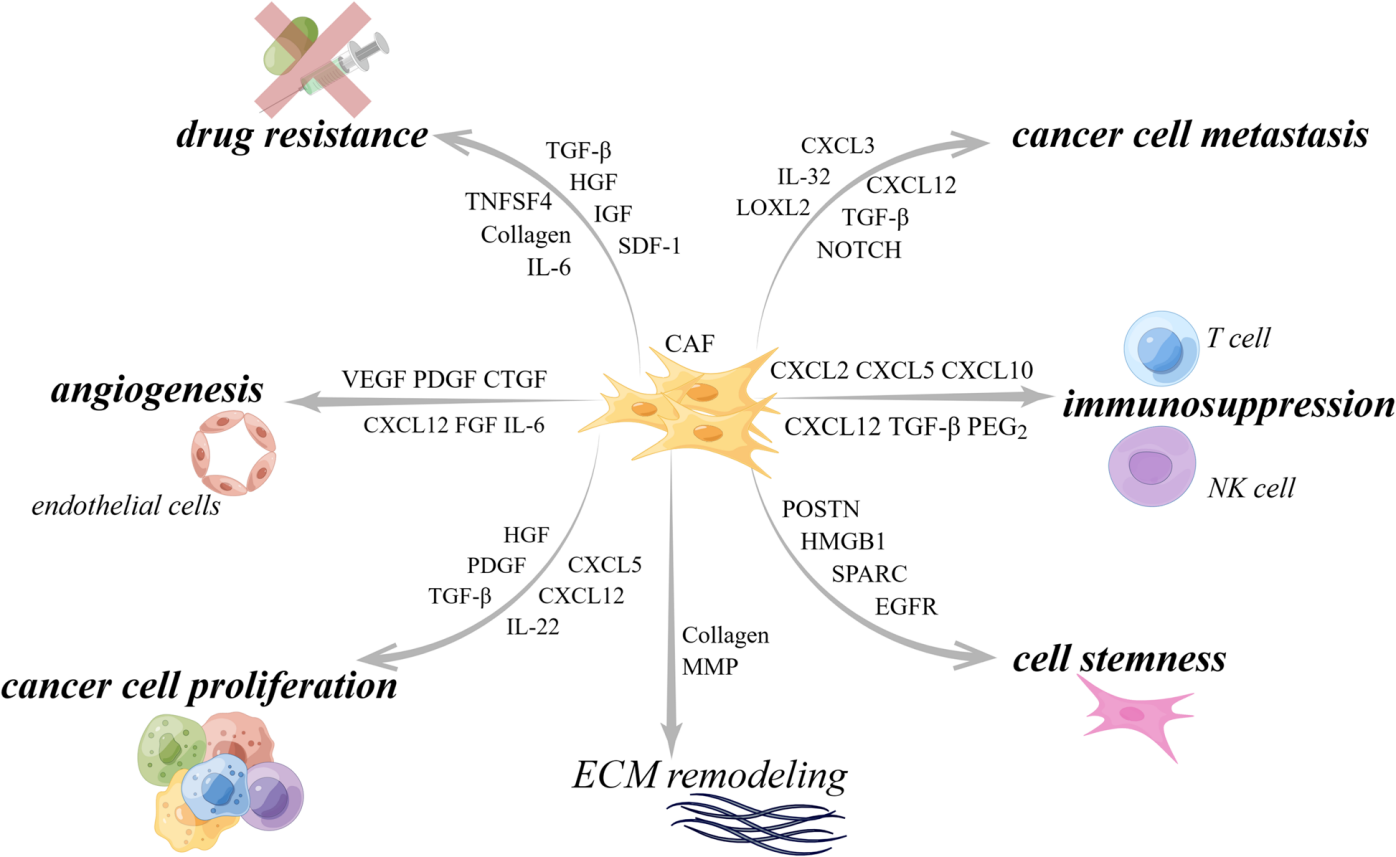
Functional heterogeneity of CAF. CAF is broadly classified as pro-tumor CAF and tumor-suppressing CAF, both of which affect tumor progression through multifaceted mechanisms. However, there are still other potential functions that have not been discovered, and it is not yet possible to determine whether this function is beneficial or harmful to tumor progression. ECM, extracellular matrix; SHH, Sonic Hedgehog. By Figdraw

As one of the major components of TME, CAFs have been shown to interact with tumors by multiple mechanisms: inducing tumor cell proliferation [9], affecting tumor angiogenesis [10], shaping immunosuppressive microenvironment to escape from immune surveillance [100], and promoting tumor formation and drug resistance [11]. For the above-mentioned reasons, CAFs are historically considered imperative tumor-promoting components. However, as a result of intensive research, much evidence supporting the tumor-inhibitory effects of CAFs has emerged, suggesting that the role of CAFs is not singularly promotional or inhibitory, but rather falls somewhere in the middle. For instance, the depletion of αSMA^+^ myofibroblasts in PDAC suppressed tumor immune surveillance with an increase in the percentage of regulator T cells (Treg, CD4^+^Foxp3^+^), which led to aggressive tumor progression and reduced animal survival [101]. In addition, Bhattacharjee et al. discovered that myCAF-expressed type I collagen had a tumor-restraining role in PDAC and colorectal cancer (CRC) metastasizing to the liver, it suppressed tumor growth by mechanically restraining tumor spread [13]. Consistent with the previously mentioned, Chen et al. deleted type I collagen in αSMA^+^ myofibroblasts in pancreatic cancer (PC) mouse model, significantly reducing the OS of mice and accelerating PDAC progression [102]. These studies demonstrate that some CAF subpopulations have tumor suppressor effects to some extent. On the basis of current findings, CAFs can be described as a group of cells with functional heterogeneity (Fig. 2). Research and work are urgently entailed to elucidate the clinical relevance of CAF heterogeneity. Below, we will elaborate on the crosstalk mechanisms between CAF and tumor components.

#### The cancer-promoting functions of CAFs

Accumulating evidence continues to signify that CAFs have pleiotropic pro-tumor functions, including tumor cell proliferation, tumor angiogenesis, tumor invasion and metastasis, drug resistance, etc.

#### Facilitating proliferation

Persistent proliferation is one of the quintessential malignant phenotypes of cancer cells. Cancer cells can stimulate proliferation through autocrine and interact reciprocally with other cells in TME to form feedback signals to promote proliferation [103]. Among them, the cross-linking between CAF and cancer cells is extensively reported. Glucose, amino acids, lipids, etc., are the material foundation of cell proliferation. However, CAF was found to change and reprogram the behavior of metabolism of the above substances in tumor cells and directly provide nutrients to them. Intriguingly, CAF can also impact cancer metabolism through secreting exosomes. As a molecular sponge of miR-330-5p in BC cells, exosomal long noncoding RNA (lncRNA) SNHG3 can suppress mitochondrial function, expedite glycolysis, and enhance breast tumor cell proliferation [104, 105]. Some evidence suggested that the prostaglandin E2 (PGE_2_) pathway expressed by CAF highly correlates with the proliferative process. In the neuroblastoma xenograft model, a remarkable reduction in tumor cell proliferation was observed by immunohistochemical staining after inhibition of the PGE_2_ pathway by microsomal prostaglandin E synthase-1 (mPGES-1) inhibitors [106, 107]. It is noteworthy that PGE_2_ signaling is contradictory in promoting proliferation and metastasis. Elwakeel, E. et al*.* observed growth inhibition of primary tumors in mice after knocking out prostanoid E receptor 3 (EP3) restriction PGE2 signaling in CAF. Still, the induction of metastatic features of tumor cells and the regulation of CAF phenotypes were also investigated [107]. CXCL12/CXCR4 cascade in FAP^+^CAF also contributed to cancer cell proliferation [108]. In addition, CAF-expressed methyltransferase NNMT in tumor stroma can support ovarian cancer proliferation. It becomes a potential therapeutic target because of its multifaceted metabolic regulatory functions, including cancer progression and CAF differentiation [109].

#### Potentiating angiogenesis

CAF has been reported to contribute to tumor angiogenesis through VEGF-dependent and VEGF-independent pathways [110–112]. In PDAC, scRNA-seq analysis technology has been used to confirm that CAF overexpresses several proangiogenic factors, supporting the pro-angiogenic effect of CAF [101]. CAFs produce angiogenesis regulators, such as VEGFA, PDGFC, FGF2, CXCL12, osteopontin, and CSF3 to promote the growth of tumor-associated blood vessels by recruiting myeloid cells and accelerate tumor angiogenesis by attracting vascular endothelial cells and recruiting monocytes [5, 49, 113]. CAF can also increase the formation of vascular mimicry (VM), and the contact between cancer cells and CAF via the Notch2-Jagged1 pathway contributes to the formation of VM networks. Simultaneously, the formation of VM was associated with anti-VEGF treatment resistance, and the combination treatment with anti-VEGF antibody and γ-secretase inhibitor DAPT, which can inhibit the Notch signaling, significantly restrained the growth of lung cancer [114]. In addition, the deletion of connective tissue growth factor (CTGF) belonging to the CCN family has been shown in melanoma studies to affect CAF activation and neovascularization, suggesting that CAF-derived CTGF is highly correlated with tumor angiogenesis. At the same time, CAFs-secreted CTGF has also been found to be associated with a poor prognosis for malignant mesothelioma, promoting metastasis [115–118]. Subsequently, Chitinase 3-like 1 (CHI3L1) secreted by CAFs acts on CAFs to increase IL-8 secretion and promote angiogenesis in CRC [119].

#### Promoting invasion and metastasis

CAFs also exert their pro-tumor function by affecting tumor metastasis. Fibronectin (Fn) is a large outer cell membrane protein found on the surface of various animal cells. Fn plays a vital role in cell adhesion, regulating cell polarity and differentiation. CAFs align Fn by increasing contractility and traction, promoting directed migration of prostate and pancreatic cancer cells, which are mediated by α5β1 integrins and PDGFRα [120]. It is well described that in BC, distinct amounts of S1 CAFs and S4 CAFs were found in metastatic breast cancer axillary lymph nodes, conducting tumor cell migration and invasion via CXCL12, TGFβ, and NOTCH signaling pathways, respectively [89, 121, 122]. The overexpression of RHBDF2 activated by TGFβ1 signaling can be observed in CAFs isolated from human diffuse-type gastric cancers (DGC), which can enhance the motility of CAF, and the highly active CAF, in turn, helps DGC cells to invade [123]. Moreover, Daniel, S. and colleagues indicated that CAFs promote GC cell survival and metastasis via activating CXCL12/CXCR4 axis. GC cell invasion was inhibited after CXCR4 antagonist (AMD3100) treatment, indicating that targeting CXCL12/CXCR4 might be a promising therapy in clinical treatment [124, 125]. Hemalatha, S. K. et al*.* have demonstrated that the conversion process from CAF to Metastasis Associated Fibroblasts (MAFs), a type of cell associated with the metastasis process, can be mediated by cancer cells, further promoting cancer metastasis [126, 127]. Additionally, PDAC metastasis was reported to be induced by myoCAF through type III collagen hyperplasia via the IL-33-ST2-CXCL3-CXCR2 axis. Heparan sulfate proteoglycan 2 (HSPG2) or perlecan, whose pro-metastasis function was identified, was observed more expression in metastatic CAFs than in weakly metastatic cancer. Intriguingly, primary CAFs named mutant-educated CAFs isolated from KPflC and KPC mice established a microenvironment conducive to invasion [128–131]. Of note, several cytokines derived from the CAF display confirm its pro-metastasis features. For instance, CXCL5, regarded as an invasive phenotype of tumor cells, can indirectly facilitate tumor growth. According to Zhou, S.-L, CXCL5 exacerbated intrahepatic cholangiocarcinoma (ICC) progression and metastasis by recruiting intratumoral neutrophils [5, 132, 133]. Another CAF-secreted chemokine, CCL5, can induce metastasis of hepatoma cells, which was achieved by inhibiting hypoxia-inducible factor 1α (HIF1α) degradation, thereby upregulating the gene zinc finger enhancement protein 1 (ZEB1) and inducing EMT [134]. Microfibrillar-associated protein 5 (MFAP5) was reported to facilitate the proliferation and invasion of bladder cancer cells in vivo and in vitro experiments [135]. In addition, fibroblast growth factor-2 (FGF2) was observed to promote BC cell migration and invasion through the paracrine FGF2-FGFR1 circle [136]. More importantly, CAFs-derived interleukins are essential in tumor progression and metastasis. IL-6, abundantly expressed in tumors, can protect gastric cancer cells through paracrine signaling and promote the invasion of BC cells. On this account, the hidden mechanism entails deeper exploration. What’s more, IL32 promotes the invasion and metastasis of BC cells through the integrin β3-p38 MAPK signaling pathway [137]. IL33 has been shown to facilitate lung metastasis in BC via instigating type 2 inflammation [138].

ECM is a complicated network comprising diverse extracellular-secreted macromolecules. The major compositions of ECM are glycoproteins, proteoglycans (PGs), and fibrous proteins like collagens and elastins [139, 140]. ECM is best described as the environment in which cells can develop [141]. Normal fibroblasts are embedded in the fibrillar ECM of the interstitium and do not associate with the basement membrane [7]. Whereas CAFs play an essential role in remodeling ECM. CAFs can synthesize ECM proteins and ECM-remodeling enzymes. The magnificent ECM biosynthesis and deposition ability of CAFs makes neoplastic tissues stiffer than normal tissues. Matrix metalloproteinases (MMPs), a family of zinc-dependent endopeptidases and one of the ECM-degrading proteases, were first described by Gross and Lapiere in 1962. The production of MMPs allows CAFs to degrade the ECM, further facilitating cancer cell invasion and making MMPs viable cancer targets. In lung cancer, the increase in tumor tissue stiffness can be attributed to the remodeling of the ECM and the secretion of growth factors by CAFs, which improve the attachment of metastatic cancer cells to the tumor endothelium, thereby exacerbating the progression of metastatic tumors. Moreover, large amounts of deposited ECM can exert a protective function via upregulating programmed death-1 receptor-ligand (PD-L1) expression in lung cancer cells [139, 142–144]. Nguyen, E. V. et al*.* revealed that CAF-secreted lysyl oxidase-like 2 (LOXL2) could expedite ECM alignment, which was conducive to the migration of prostate CAF and cancer cell [142, 145].

#### Drug resistance

Multiple findings validated that CAFs can contribute to chemotherapy and radiotherapy resistance through numerous mechanisms, which led to therapeutic failure. Conversely, CAF can enhance tumor cell resistance by directly secreting cytokines and delivering exosomes. The previously mentioned CAF subtype expressing inflammatory factors in melanoma inhibited immune-checkpoint blockade (ICB) therapy response, and CAF-secreted CXCL12 contributed to tumor progression and gemcitabine resistance via upregulating SATB-1 secretion [91, 146]. P35 was a vital cancer suppressor gene, and IL-6, secreted by CAF, has been reported to exert a protective effect on cancer cells. IL-6 attenuated the p53 response via the JAK/STAT pathway, inhibited doxorubicin-induced cell death, and increased the survival of prostate cancer cells [147, 148]. Still, in prostate cancer, CAF-derived exosomes miR-423-5p inhibited the GREM2 (Gremlin 2) gene via the TGF-β pathway, increasing resistance to taxane. The exosome miR-22 secreted by CD63 CAFs can bind to ERα and PTEN, and confer tamoxifen resistance in BC cells. Furthermore, CD63 neutralizing antibodies counteracted these responses, suggesting that CD63 CAF may be a possible target to restore sensitivity to tamoxifen therapy [149]. Alternatively, exosome LINC00355 has been demonstrated to promote cisplatin resistance of bladder cancer cells via the miR-34b-5p/ABCB1 axis. Previous studies have shown that exosomal LINC00355 can facilitate the proliferation and invasion of bladder cancer cells as well [150–152]. Apart from this, Fang, Y. and colleagues found that CAF had endogenous resistance to gemcitabine compared to NF. CAF also delivered miR-106b directly to pancreatic cancer cells via exosomes, targeting the TP53INP1 gene to promote GEM resistance in cancer cells [153]. On the other hand, CAF boosted drug resistance by interacting with other TME cellular components. A study by Haldar, S. and colleagues reported that the synergistic effect of docetaxel and C3aR could impair the mitochondrial DNA (mtDNA) /C3a paracrine loop, restore the sensitivity of prostate cancer (PCa) cells to taxanes, and inhibit tumor expansion. Mechanistically, the mtDNA secreted by the PCa epithelium binds to the transmembrane protein DEC205 on the surface of CAFs, activating TLR9 and the maturation of the allergic toxin C3a, which enters TME and favors tumor cell proliferation and insensitivity to docetaxel [154]. A tumor immune barrier (TIB) formed by crosstalk between SPP1 macrophages and CAFs created an immunosuppressive microenvironment that hindered peripheral tumor infiltration of immune cells such as CD8^+^ T cells, thereby suppressing immunotherapy efficacy in hepatocellular carcinoma (HCC). Specifically, targeting SPP1 macrophages reduced the aggregation of CAF, again demonstrating the interaction between them [155]. Additionally, G-protein-coupled receptor 30 (GPR30) activated in CAF upregulated the expression and secretion of high mobility group protein 1 (HMGB1) in CAF. The overexpressed HMGB1 triggered the MEK/ERK signaling pathway and induced autophagy, which enhanced MCF-7 cell resistance to tamoxifen, thereby sparing BC cells from tamoxifen-mediated apoptosis [156]. As reviewed elsewhere, CAF can also bestow drug resistance upon tumor cells by regulating metabolism and inducing epigenetic modifications [157, 158]. More recent findings suggested that ferroptosis may be involved in the treatment resistance as well. Ferroptosis was first proposed by Dr. Brent R.Stockwell in 2012 as a new manner of non-apoptosis, non-cellular necrosis, and iron-dependent cell death. The essence of ferroptosis is the inactivation of glutathione peroxidase, which leads to the accumulation of lipid peroxidation. Recently, researchers have discovered that CAF can inhibit ferroptosis in tumor cells through specific pathways. In GC, miR-522 secreted by CAF was a potential inhibitor of arachidonate lipoxygenase 15 (ALOX15), which was closely associated with toxic lipid peroxides. More importantly, this study demonstrated that paclitaxel and cisplatin could promote CAF secretion of miR-522 through the USP7/hnRNPA1 pathway, reducing chemotherapy sensitivity and revealing a new mechanism of chemotherapy resistance [159]. Similar results were found in glioblastoma (GBM). CAF upregulated the expression of lncRNA DLEU1 by activating HSF1, conferring ferroptosis resistance to GBM cells [160]. At last, it is also noteworthy that ECM deposition pertains to drug resistance. In PDAC, more than half (sometimes to 80% of the tumor mass) of the neoplastic tissues are composed of stromal tissues secreted by CAFs and other components. The hardened ECM can form a physical barrier, which hinders the arrival of chemotherapy and immunotherapy drugs to the cancer site via compressing peripheral blood vessels to reduce blood flow, thus attenuating the efficiency of drug delivery [6, 161, 162].

#### Immunosuppression

To survive and proliferate, CAFs must find ways to evade the immune system's surveillance at the cancer site. Although the intricate underlying mechanism of CAF suppressing immunity has not been fully understood, many studies have shown that CAF can suppress immunity in diverse ways. By secreting cytokines and chemokines like TGF-β and CXCL12, CAFs prevent the activation and recruitment of T lymphocytes in cancer sites [163, 164]. Importantly, CXCL12 exerts its anti-inflammatory function in TME by inducing the transformation of T cells into Tregs, promoting the generation of macrophages that promote angiogenesis and dendritic cells (DCs) that are poorly functioning. Besides, the CXCL12/CXCR4 axis has been identified as associated with immune suppression and metastasis via recruiting immunosuppressive cells in numerous solid tumors. Recently, a study unraveled that a ketogenic diet (KD) has increased natural killer (NK) cell and cytolytic T lymphocyte (CTL) infiltration while improving immunosuppression by repressing CXCL12 in CRC. Mechanistically, KD significantly reduces the expression of KLF5 via increasing ketogenesis by overexpressing ketogenic enzyme 3-hydroxy-3-methylglutaryl-CoA synthase 2 (HMGCS2), which attenuates CXCL12 expression in CAF through binding to the CXCL12 promoter [164–167]. Of note, the TGF-β pathway can also directly promote the growth of CAFs, further influencing cancer progression. The overexpression of TGF-β can elicit CAF formation [168]. TGF-β1 can induce normal fibroblasts into CAFs in bladder cancer, and CAF proliferation has been significantly attenuated after using a TGF-β receptor inhibitor [169, 170]. Besides, TGF-β1 affects EMT and invasion of BC cells through CAFs activation via overexpressing FAP and autophagy [171]. CAF-S1, a subset of myofibroblast, recruits CD4^+^CD25^+^ T cells to create an immunosuppressive microenvironment via CXCL12 and expresses B7H3, CD73, and DPP4 to promote their differentiation into Tregs, thereby contributing to tumor growth [98]. Notably, the accumulation of myeloid-derived suppressor cells (MDSC) is considered a signal of increased immunosuppression. CAFs were reported to induce the differentiation of monocytes into MDSC via IL-6-mediated signal transducer and activator of transcription 3 (STAT3) activation manner [122, 172–174], and FAP^+^CAFs can recruit MDSCs infiltration via STAT3-CCL2 signaling. Thus, it is persuasive that STAT3 hyperactivation can provide favorable conditions for CAFs to create an immunosuppressive microenvironment. More evidence indicated that in vitro and in vivo mouse BC models, CAF-intrinsic STAT3 activity exerts pro-tumorigenic functions through STAT3-dependent mediators like ANGPTL4, MMP13, and STC-1 [175–177].

The crosstalk between CAF and immune cells is gradually being unveiled. According to Kato T. et al*.*, CD8^+^ T lymphocytes and CAFs were negatively correlated in intratumoral tissues [178]. CAFs create an immune barrier to CD8^+^ T cell-mediated anti-tumor immune responses. It has been verified that CAF can diminish CD8^+^ T cell infiltration in tumors and contribute to ICB resistance [179, 180]. CAFs even directly kill CD8^+^ T cells in an antigen-specific manner via PD-L2 and FASL [181]. In bladder cancer, FAP^+^ CAFs were associated with poor infiltration of CD8^+^ T cells with stromal changes and significant loss of human leukocyte antigen (HLA-I) expression in cancer cells. Similar results were observed in HCC as well. Researchers showed that CAFs and M2 macrophages might pertain to CD8^+^ T cell exhaustion in steatotic HCC [182]. Another recent study found that apCAFs induced naive CD4^+^ T cells into Tregs, which disturbed the growth of CD8^+^ T cells in pancreatic cancer via IL-1 and TGFβ signaling pathways [183]. In stage-I lung squamous cell carcinoma (SqCC), PDPN^+^ CAFs highly expressed TGF-β1 and recruit immunosuppressive cells like CD204^+^ tumor-associated macrophages [184–186]. Besides this, CAFs directly enhanced the recruitment of pro-tumoral immune cell populations, manifested by an increased Th2 response and a decreased Th1 response [187]. Th1 cells participated in the defense of the body from intracellular pathogens. By secreting TNF-α, TH1 cells inhibited the occurrence and development of tumors. Moreover, CAFs can highly express immune checkpoint ligands like PD-L1. PD-L1 suppresses anti-tumor immunity by binding to the receptor PD-1 on activated T lymphocytes to counteract T cell activation signals [187, 188]. Similar results were reported by Dou D. and colleagues. CAF-derived exosome microRNA-92 increased the expression of PD-L1 in BC cells, which was correlated with impaired T cell proliferation. Animal studies conducted by the same group further confirmed the functional impairment of tumor-infiltrated immune cells in vivo [189]. In addition, four CAF subtype populations were identified in NSCLC by paired scRNA-seq and IHC analysis. In tumor lesions containing MYH11^+^αSMA^+^ CAF and FAP^+^αSMA^+^ CAF, the density of CD3^+^ or CD8^+^ T cells was remarkably reduced compared to T cell-permissive CAFs, indicating that both CAFs were associated with T cell exclusion [190].

**Fig. 3:**
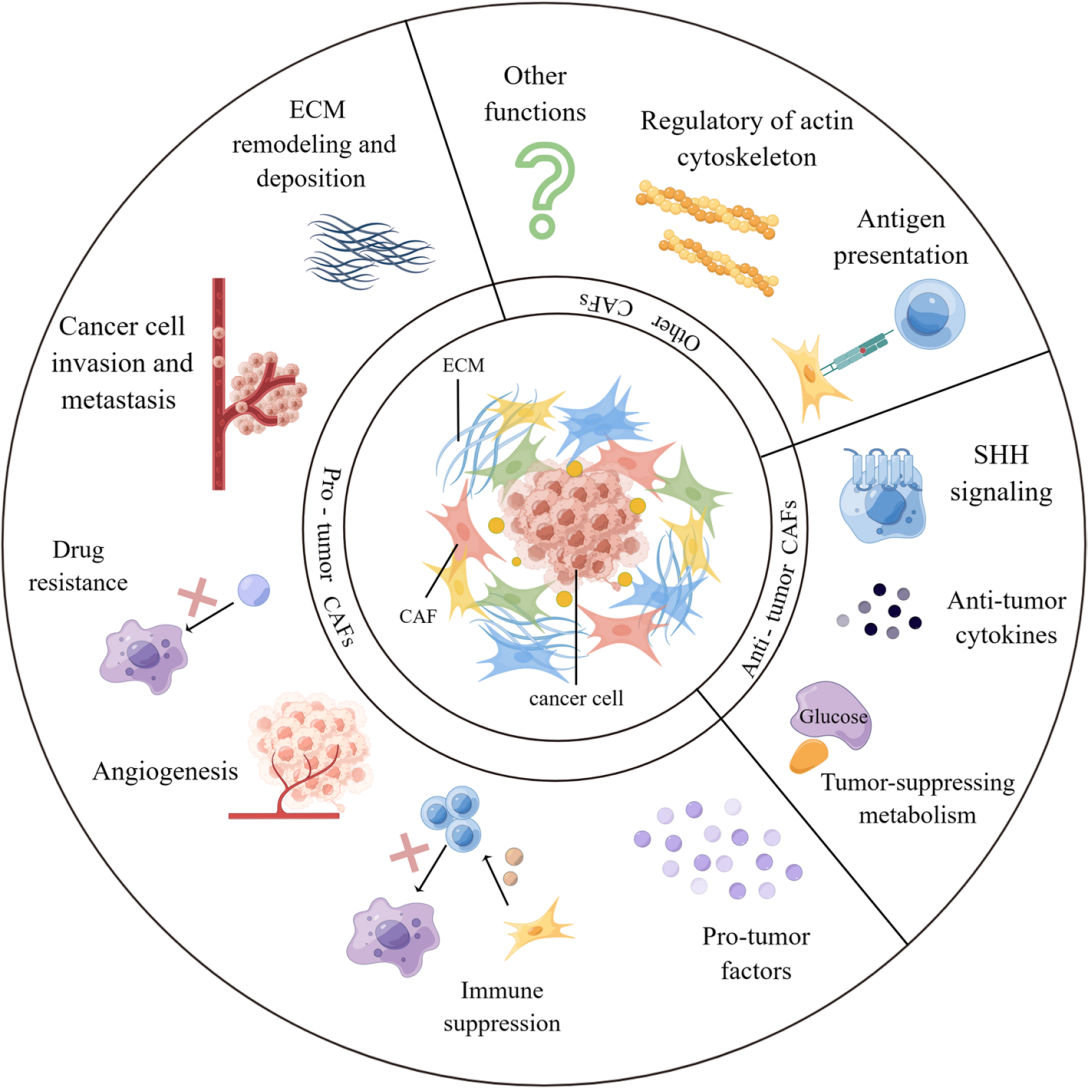
CAF interacts with a variety of tumor-promoting components through multiple signaling pathways. CAF can exert its pro-tumor function by promoting tumor neovascularization, promoting tumor cell proliferation and metastasis, regulating tumor microenvironment to an immunosuppressive state, and reconstructing ECM, etc. CAF, cancer-associated fibroblast; CTGF, connective tissue growth factor; CXCL, C–X–C motif chemokine; EGFR, epidermal growth factor receptor; FGF, fibroblast growth factor; HGF, hepatocyte growth factor; HMGB1, high mobility group protein 1; IGF, insulin-like growth factor; IL, interleukin; NOTCH3, neurogenic locus notch homolog protein 3; LOXL2, lysyl oxidase like 2; MMP, matrix metalloproteinase; PDGF, platelet-derived growth factor; PGE2, prostaglandin E2; POSTN, periostin; SDF-1, super dimensional fortress-1; SPARC, secreted protein acidic and cysteine rich; TGF-β, transforming growth factor beta; TNFSF4, tumor necrosis factor superfamily member 4; VEGF, vascular endothelial growth factor. By Figdraw

Overall, existing studies have explored the pro-tumor function of CAF in multiple ways. In practice, the detailed mechanisms responsible for the biological pro-tumor role of CAFs still need to be discussed meticulously. Nevertheless, the contribution of CAFs to tumor progression mentioned above is just the tip of the iceberg, multiple signaling pathways were demonstrated to pertain to the pro-tumor functions of CAFs (Fig. 3). New research from Sazeides, C. & Le, A. suggests that exosomes derived from CAFs (CDEs) contribute to reprograming cancer cells' metabolic activity via downregulating specific genes [184, 191]. Moreover, cancer stem cells (CSCs) were found to be regulated by TME components like CAF. CAFs maintain transfer colonization of CSCs via periostin. BCSCs also express the Hh ligand Shh, which enables CAF’s expansion through paracrine. In PC, researchers revealed that CAFs could facilitate cancer stemness via the OPN / SPP1-CD44 axis, and the promoting effects were counteracted after a specific blockade. Even so, the interactions between CAFs and CSCs have yet to be discovered [192, 193]. Still, most of the experiments to validate the cancer-promoting functions of CAF were done in xenotransplantation or co-implantation models, which might cause errors or deviations in the transcriptional process of CAF biomarkers. Consequently, the fundamental mechanisms of CAFs in human tumors remain to be confirmed.

#### The cancer-restraining functions of CAFs

In line with the functional heterogeneity of CAF mentioned earlier, notwithstanding the majority of the existing studies have focused on the various pro-tumor functions of CAFs, the inhibitory functions of CAF on cancer should not be neglected and remain to be elucidated in more detail. Recent experiments have detected the expression of CD9, CD63, and CD81 in CAF-derived exosomes by western blotting. Surprisingly, CD9-positive exosomes can inhibit the proliferation of malignant melanoma. This study also highlighted that patients with CD9-positive exosomes showed longer five-year survival rates [194]. Evidence from recent studies has also demonstrated that CAF can remarkably improve drug sensitivity. In lung cancer, a particular subset of CAF: CD200-positive CAF was uncovered to elevate the sensitivity of cancer cells to EGFR-tyrosine kinase inhibitor (EGFR-TKI), gefitinib, and the sensitizing potential was deprived when CD200 was knocked out [195]. Another study unraveled that when CAF was co-cultured with NSCLC cells, the secretion of IGF and IGF-binding proteins (IGFBPs) was linked to the drug sensitization of EGFR-TKI [196]. As mentioned, deleting αSMA^+^ myCAFs in PDAC increases immunosuppression and reduces OS. A previous study has reported that CAFs can impede PDAC progression by hypoxia. Meanwhile, according to Rhim, A. D. et al*.*, Sonic hedgehog (Shh) is a soluble ligand overexpressed in PDAC tumor cells that promotes the formation of the fibroblast-rich stroma. Deleting SHH in murine models did reduce the interstitium of tumors, but at the same time, SHH-deletion tumors also showed more extraordinary proliferative ability and aggressiveness. In a way, myCAFs exert tumor suppressor function partially through the SHH-SMO signaling pathway [5, 7, 12, 197, 198]. Simultaneously, Bhattacharjee and colleagues have demonstrated that myCAF-expressed type I collagen can physically restrict desmoplastic tumor growth. They found that deletion of type I collagen in mice models tremendously promoted tumor metastasis in PDAC and CRC. A possible mechanism is that type I collagen establishes a mechanical barrier that limits tumor growth [13]. Again, Tanaka, R. and colleagues recently revealed that CAF-secreted IL-8 had a suppressive effect on the proliferation of OCUCh-LM1 cell lines associated with tumor formation [199, 200]. Since apCAFs have been reported to present antigens to CD4^+^ T cells and are therefore believed to be involved in the anti-tumor process, more evidence is anticipated to fully unravel its anti-tumor role [92]. Cumulatively, the above evidence indicates that CAF also has a potential anti-tumor function that should not be overlooked.

#### Advances in targeted CAF strategies in different cancers

Collectively, the multifaceted tumor-promoting functions that CAFs exhibit during tumor progression make them appealing therapeutic targets for oncotherapy. The easiest way to target CAF is to eradicate CAF or make it functionally impaired. Endo180 is a circulating endocytosis receptor expressed exclusively in fibroblasts, with higher expression in CAF populations than in normal fibroblasts. Studies have shown that tumor growth and progression were immensely limited in Endo180^−/−^ mice due to genetic deletion of the Endo180 receptor. This pro-tumor functional damage was caused by CAF intrinsic contractility defects and decreased CAF activity [180, 201, 202]. One major approach to eliminate CAFs is to target specific surface markers. For instance, FAP is expressed on a subset of CAFs in different tumors. Chimeric antigen receptor T cell treatment (CAR-T) can explicitly target CAFs. From previously published studies, FAP-specific CAR-T cells can kill most FAP^+^ cells, including CAFs, and prevent the growth of tumor stroma, which enhances the absorption of chemotherapy drugs and has anti-tumor benefits. FAP-expressing cells in the tumor microenvironment have been specifically and directly removed using infrared photoimmunotherapy (NIR-PIT), a new and novel method to remove CAFs. This approach inhibited tumor growth in a co-cultured human esophageal squamous cell carcinoma xenograft model without adverse effects. So far, the FDA (Food and Drug Administration) has approved five CAR-T therapies for hematological malignancies of B cell origin. In contrast, no CAR therapy has been approved for solid tumors yet [203]. Nevertheless, obtaining clinical benefits is not necessarily limited to completely eradicating or reprogramming CAF but can be achieved by blocking signals from CAF. In that signaling pathways are partially related to CAF, some promote the growth, proliferation, invasion, and metastasis of CAF through the secretion of various factors. Other pathways expressed by CAF modulate or transform the TME to make it generally conducive to tumor growth. Moreover, numerous anticancer medications undergoing human testing may also target CAF or its metabolites. Histone deacetylase (HDAC) and SMO inhibitors have undergone extensive testing in numerous clinical trials. These medications alter intracellular signaling and epigenetic regulation in tumor cells, CAFs, and CAF precursors [204]. It is important to note that CAF.ERα( +) (estrogen receptor alpha) can impede the metastasis and invasion of prostate cancer by inhibiting macrophage infiltration and modulating the expression of thrombospondin 2 (Thbs2) and MMPs [205, 206], which emphasizes the need for caution in targeting CAF. As our understanding of CAF biology in cancer deepens, CAF-targeted therapies are gradually being reinvigorated, and many clinical trials are underway. Next, we will comprehensively introduce advances in targeting CAFs in several types of cancer.

#### Breast cancer

Breast cancer, a malignant tumor that seriously endangers women's health and is occasionally seen in males, has become a public health issue worldwide [207–209]. In 2020, it was the most diagnosed malignancy [210]. According to the difference in the expression level of different hormone receptors: ERα (estrogen receptor α), PR (progesterone receptor), and HER2 (human epidermal growth factor receptor 2), BC can be briefly classified into four types: luminal A, luminal B, HER2‐positive, and triple‐negative, and of course, the prognosis of each varies [122, 211]. Today's main treatments for breast cancer are radiotherapy, chemotherapy, endocrine therapy, surgery, or a combination of these. Despite all the progress made in the past decade, the incidence rate of BC has risen continuously. Targeting CAF therapy may shed light on the current clinical BC treatment.

#### Targeting CAFs specific molecules and biomarkers

As a significant biomarker of CAFs and an emerging cancer promotor, FAP is deemed one of the most feasible and clinically useful CAF markers. Thus, innumerable studies have been designed to look into FAP in recent years. Administration of an anti-FAP monoclonal antibody (mAb), FAP5-DM, has provided long-lasting inhibition of tumor growth and even complete tumor regression with no signs of intolerability in stroma-rich xenograft models of various cancers [212]. In another mouse 4T1 metastatic BC model, researchers developed a FAP-targeting immunotoxin αFAP-PE38 to deplete FAP-positive stromal cells, which showed efficacy in suppressing tumor growth [213]. Of note, FAP-targeted vaccines have shown their antitumor function in both in vitro and in vivo experiments, modulating the immunosuppressive microenvironment and decreasing tumor growth and angiogenesis [122, 214]. To date, several FAP-based vaccines have been investigated in preclinical trials. Administration of oral FAP DNA vaccine induced CD8^+^T cell–mediated killing of CAFs and successfully suppressed primarytumor growth and colon and breast carcinoma metastasis in multidrug-resistant murine models. DNA vaccine can remarkably decrease stroma type I collagen expression and improve the efficacy of chemotherapy [215]. Recently, a synthetic consensus (SynCon) FAP DNA vaccine has displayed superiority at breaking immune tolerance compared to the native FAP immunogen in genetically diverse mice. The SynCon FAP DNA vaccine synergized with other tumor-antigen-specific DNA vaccines showed a stronger anti-tumor activity than monotherapy, and the SynCon FAP DNA vaccine itself exerted remarkable antitumor effect in the TC-1, Brpkp110, and TSA tumor models [216]. So far, the use of DNA vaccines has been limited to animal experiments, and no DNA vaccines have moved into the clinic. Researchers have developed an FAP.291-based epitope minigene vaccine that can activate CTL against CAFs and suppress tumor progression in murine BC models [217]. Moreover, several drugs targeting FAP have been submitted to clinical trial-enrolled patients with metastatic CRC, including Sibrotuzumab (a FAP targeting humanized monoclonal antibody) and Talabostat [218, 219]. However, all these drugs failed to pass clinical phase II trials. As reviewed elsewhere, targeting FAP molecular imaging is also booming in diagnostic imaging. For instance, in PET (positron emission tomography)/CT (computed tomography), ^68^Ga-FAPI-04, one of the quinoline-based FAP inhibitors (FAPIs) developed by the University Hospital Heidelberg, has become a more promising tracer that can discriminate cancerous lesions more accurately compared with ^18^F-FDG in a cohort of 48 BC patients. At the same time, the FAPI series has certain limitations in tumor retention. Consequently, a compound FAP-2286 was developed to overcome the obstacle, and ^68^Ga-FAP-2286 has demonstrated its ability for imaging in preclinical models, not just in BC [57, 220–222]. Except for being tumor-promoting, FAP was also observed to have some tumor-inhibiting properties. A second independent observation found that more abundant FAP of invasive breast ductal carcinoma is associated with longer overall and disease-free survival [223]. These studies confirmed the functional heterogeneity of CAFs, which was probably related to the failure of clinical trials mentioned before. It is noteworthy that CAFs do not exclusively express FAP, so the shortage of CAF-specific biomarkers greatly hindered the precision targeting of CAFs via the abovementioned approaches. Therefore, other FAP-expressing cells may also be influenced when using FAP-specific strategies to delete CAFs, leading to adverse consequences. For instance, due to the killing of multipotent bone marrow cells that express low levels of FAP, FAP CAR-T cells induced significant cachexia and lethal bone toxicities in mouse strains bearing a variety of subcutaneous tumors [224]. Thus, finding biomarkers exclusively expressed in CAFs is imperative for CAF-targeted oncotherapy. Alternatively, about 20% ~ 30% of BC patients' tumors are HER-2 positive type. The HER2-positive subtype, characterized by ERBB2 amplification, has a poorer clinical prognosis than HER2-negative tumors and is prone to recurrence [225, 226]. Anti-HER2 mAbs like trastuzumab and pertuzumab are one of the main therapeutic agents in first-line therapy. However, half of the HER2-positive patients benefit little to no from HER2-targeted therapy, and one in five patients will relapse after treatment. Studies have highlighted that CAFs play an essential role in the anti-HER2-targeted therapies resistance. Rivas, E. I. et al*.* revealed that aggregation of CAFS 1 and pCAF in the CAF subtype of BC was significantly increased in patients who did not respond to anti-HER 2 mAb therapy, potentially leading to reduced IL2 activity. In contrast, low IL2 activity may be associated with treatment resistance. Besides, FAP is the biomarker expressed by both CAF S1 and pCAF, they found that IL2 activity was maintained using a novel immunocytokine FAP-IL2v, Simlukafusp Alfa. In vitro models, this monoclonal antibody fusion protein consisting of an IL-2 variant and a FAP-targeting protein has been shown to enhance antibody-dependent cellular cytotoxicity (ADCC) by activating NK cells. It is currently undergoing evaluation in a phase I clinical trial in combination with trastuzumab. In addition, in murine models of multiple human cancers, FAP-IL2v combined with various therapeutic antibodies has also shown some positive efficacies [226–228].

#### Targeting CAF-associated signaling pathways

Targeting CAF-associated signaling pathway therapy should not be dismissed, the TGF-β signaling pathway has attracted the attention of oncologists in the past decade. The links between TGF-β and CAF include (1) CAF paracrine TGF-β can induce EMT of breast cells, promote the transformation of BC cell lines to a more invasive phenotype, and activate the TGF-β/Smad pathway. CAFs are reported to activate the transcription of HOTAIR through TGF-β1 secretion to promote BC cell metastasis; (2) the autocrine TGF-β1/miR-200 s/miR-221/DNMT3B loop maintains CAF activity and promotes BC progression, and destroying the loop can restore the NF phenotype; (3) TGFBR2 expressed by CAF affects the growth and survival of BC cells; (4) the elevated level of TGF-β transcription in BC stimulates the conversion of NFs to CAFs, and gene ZNF32 prevents NF-to-CAF conversion by directly binding to the TGFB1 promoter to inhibit the transcription process; (5) the hyperactivity of TGF-β signaling pathway in CAF is often associated with immunotherapy failure [229–234]. Therefore, a number of TGF-β pathway inhibitors were developed. Fresolimumab, a neutralizing antibody that targets TGF-β1,2,3, has confirmed its anti-tumor feasibility and safety in a phase II clinical trial (NCT01401062), in which researchers focused on the cooperation of Fresolimumab and focal irradiation while applying to 23 patients with metastatic BC. Participants were divided into two groups, receiving different doses of Fresolimumab. Seven grade 3/4 adverse events occurred in 5 of 11 patients in the 1 mg/kg group and 2 of 12 patients in the 10 mg/kg group, respectively. Higher doses of Fresolimumab were shown to improve median OS, as the median OS was reported to be 7.57 months in the low-dose group compared to 16.00 months in the high-dose group. [229, 235, 236]. The combination of TGF-β receptor I kinase inhibitor Galunisertib (LY2157299) and PD-L1 blockade has also shown excellent results in tumor treatment. To date, Bintrafusp alfa (BA), a fusion protein that can simultaneously inhibit both TGF-β and PD-L1 pathway, was demonstrated to possess a stronger affinity with TGF-β1 and inhibition of cancer cell proliferation than Fresolimumab in MC38 tumors [237, 238]. In addition, targeting TGF- β1 was regarded as a method to solve chemoresistance in CAFs [239]. It is intriguing that losartan, the first angiotensin II receptor antagonist, typically known as the antihypertensive drug, can downregulate the TGF-β pathway and inactivate CAFs. Patients with triple-negative breast cancer (TNBC) are relatively resistant to anti-PD1 therapy. Zhao Q and colleagues proposed a combined therapy of Losartan, doxorubicin hydrochloride liposome (Dox-L), and α-PD1, which results in reduced ECM and better regulation of the immune microenvironment, may guide the clinical treatment regimen of TNBC. Moreover, losartan has been reported to improve delivery efficiency and the therapeutic effect of photodynamic nanoplatforms by depleting tumor collagen [240–242]. Natural compounds like Zerumbone (ZER) were found to repress BC cell metastasis via downregulating mRNA transcription. ZER has been shown to reduce the neoplasticity and motility of TNBC cells by inhibiting the TGF-β1 signaling pathway and can increase the sensitivity of BC cells to paclitaxel [243–246].

CXCR4 has been described to promote BC cell proliferation and expedite tumor growth via recruiting immune cells and facilitating angiogenesis [247]. The CXCL12/CXCR4 pathway has emerged as a vital part of BC tumorigenesis and in BC metastasis to the brain, liver, and lung in the past few years [248–250]. It is reported that the recruitment of endothelial progenitor cells (EPCs) was mediated by CAF-derived CXCL12, which promoted angiogenesis in BC, and CXCL12 secreted by CAF also directly stimulated tumor growth. Autocrine CXCL12 signaling in breast fibroblasts initiated and maintained the pro-tumor CAF phenotype [230, 251]. In addition, due to the CXCL12-CXCR4 axis driven by CAF, monocytes were recruited into tumor sites to acquire the tumor-promoting ability of lipid-associated macrophage to maintain the immune microenvironment in a suppressed state [252]. The CXCR4 antagonist AMD3100 was observed to increase CTL infiltration and reduce desmoplasia and immunosuppression in mouse metastatic BC models [253]. AMD3100 also attenuated TNBC cell migration and metastasis in zebrafish embryos [254]. Similarly, Combination therapies have shown promising results. In an animal experiment, AMD3100 and tamoxifen significantly alleviated tamoxifen resistance without obvious side effects [255]. The combination of AMD3100 and PARP1 inhibitor, Olaparib was found to have a positive correlation and can suppress tumor growth and metastasis in vivo TNBC animal experiments via inducing severe DNA damage [256]. AMD3100 combined with anti-PD-1 therapy has proven useful more than just in murine BC models [124, 257–259]. Wu, Y. et al*.* elucidated that the blockade of FGFR signaling by Erdafitinib mechanically degraded the secretion of vascular cell adhesion molecule 1 (VCAM-1) through downregulating MAPK/ERK pathway in CAFs, creating a favorable microenvironment for T cell infiltration [260]. Furthermore, CAFs were found to have close communication with BC cells via the HGF-MET pathway. Blocking HGF-MET signaling can simultaneously target primary TNBC tumorigenesis and lung metastasis in a three-dimensional organotypic tumor model and alleviate radioresistance [261, 262]. Analogically, the potential of the HGF-MET pathway as a therapeutic target was discovered in NSCLC and prostate cancer [263, 264].

As for the STAT3 signaling pathway, it has been shown that CAF-secreted TIMP-1 activated the STAT3 pathway in BC cells, promoting proliferation and migration, and CAF-derived IL-6 can increase extracellular TIMP-1 abundance, suggesting that inhibition of TIMP-1/CD63/integrin β1/STAT3 loop may be a promising therapeutic modality. In addition, CAF-derived IL-6 can directly activate the STAT3 pathway, promoting the growth and radioresistance of BC cells [265, 266]. The primary representative inhibitors can be divided into peptides, small molecules, and oligonucleotides. In pre-clinical cancer models, Peptides such as ISS-610 prodrugs and small molecules like compound 6o, Stattic, and FLLL32 were demonstrated to generally upregulate apoptosis of BC cells [267–269]. Similarly, Stattic can resensitize BC cells to tamoxifen by inhibiting cell proliferation and inducing apoptosis in tamoxifen-resistant cell lines [270]. Of note, Tocilizumab (TCZ) continuously inhibited CAF biomarkers beyond STAT3 in situ, humanized breast tumors in mice, but also reduced tumor angiogenesis and metastasis [271]. A phase 1 study (NCT03135171) enrolled 11 patients with BC to determine the safety and tolerability of the cooperation of tocilizumab, trastuzumab, and pertuzumab has recently been completed, and the result is about to be available. Alternatively, STAT3 inhibition through siRNA suppressed cancer cell proliferation and resensitized neuroendocrine tumors to mTOR inhibitor Everolimus treatment [272]. Of note, Hu, G. et al*.* unravel that CD73^+^ γδTregs was the dominant regulatory T cell in human BC and was associated with poor clinical outcomes. Mechanistically, the IL6-adenosine positive feedback loop formed between CD73^+^γδTregs and CAFs promoted the production of the immunosuppressive microenvironment and accelerated tumor progression [273]. At last, the focal adhesion kinase (FAK) signaling pathway was known for its profibrotic function and could become a drug target. Zhang and colleagues suggested that zeste homolog 2 (EZH2) activating TGF-β signaling via activating FAK signaling, using FAK inhibitors, can effectively inhibit BC bone metastasis in vivo [226, 241, 274].

#### Targeting stroma

Another target should be stroma proteins. The desmoplasia response is due to the deposition of large amounts of ECM proteins, such as fibro collagen, hyaluronic acid, and tenascin C, as well as CAF-mediated ECM reshaping. Therefore, some strategies to improve ECM stiffness, including targeting the production of ECM proteins or degrading ECM, are seen as effective protocols for targeting CAF [49, 275]. Tenascin-C (TNC) is a hexamer, multi-module extracellular matrix protein. It comes in various molecular forms and is produced by alternative splicing and protein modification [276]. TNC has been identified to regulate tumor angiogenesis and tumor immunity, especially the function of CTL, plasticity, and tumor metastasis in multiple cancers [276–280]. According to Murdamoothoo, D. et al*.*, TNC can retain CD8^+^ TIL in cancer stroma by binding CXCL12, which facilitates the progression of BC. By blocking CXCR4 with AMD3100 in murine models, CD8^+^ TIL and macrophage infiltration are promoted, causing tumor cell death [281]. Besides, in TNBC, researchers have revealed that high Tenascin-C expression correlated with poor prognosis in TNBC patients using Kaplan–Meier meta-analyses and was negatively associated with LC 3B expression and CD8^+^ T cells. Targeting TNC enables TNBC cells sensitive to checkpoint inhibitors and sensitizes PD-1 blockade therapy in mice models [282]. Several clinical trials and animal experiments on inhibiting the TNC pathway or targeting TNC and other combined factors have begun to bear fruit [283, 284].

#### Other targets of CAFs

It is also noteworthy that ceramide accumulation can upregulate the expression of tumor suppressor gene p53 and restrain CAF activation during sphingosine kinase 2 (SphK2) deletion. The utilization of SphK2 inhibitors caused ECM reprogramming, manifested by increased expression of matrix P53, restriction of fibroblast conversion to CAF, and ultimately impaired cancer progression [285]. Breast CAFs typically express the MCL-1 gene, and the MCL-1 expression level in breast CAFs is higher than in normal fibroblasts. In luminal breast cancers, MCL-1 expression is influenced by paracrine effects. Bonneaud and colleagues unraveled that targeting MCL-1 via BH3 mimetic, an antagonist to various BCL-2 congeners, including MCL-1, can mitigate the invasion of cancer cells and inhibit their tumor-promoting function. A possible mechanism is that BH3 mimetic promotes mitochondrial cleavage in bCAF [286]. Exosome-based research is in full swing, and exosome-based nucleic acid delivery has become an emerging cancer treatment option [287]. It has been suggested that inhibition of CAF-derived exosomes like miR 500a-5p and miR-92 can suppress the growth and metastasis of BC and may be a potential therapeutic target [189, 288]. Conversely, the loss of miR-4516 leads to malignancy in TNBC, suggesting that miR-4516 can potentially become an antitumor drug for TNBC [289]. In addition to drug targets, researchers have also found that CAFs and specific markers can indicate and predict prognosis. The AU-rich RNA-binding factor 1 (AUF1), Podoplanin, and ATR-negative CAFs have been shown to correlate with poor OS [290–292].

#### Pancreatic cancer

PDAC, characterized by fibroinflammatory hyperplasia, has the potential for early metastasis and accounts for more than 90% of all pancreatic malignancies. However, magnificent resistance to existing treatments such as chemotherapy, radiotherapy, and molecularly targeted therapy makes pharmacological treatment of PDAC formidable and prone to relapse. All these factors contribute to a poor prognosis for PDAC, with a five-year survival rate of less than 10% [293]. The progression of PDAC is intimately intertwined with CAFs and immunosuppressive cells such as Tregs and TAMs. Also, crosstalk between CAF and tumor cells complicates the PDAC tumor microenvironment and favors drug resistance. Therefore, novel strategies targeting CAFs have piqued researchers’ interest.

#### Targeting CAF-related proteins

The leucine-rich repeat-containing protein 15 (LRRC15), a marker of mesenchymal stem cells (MSCs) that belonged to the LRR family, was highly and exclusively expressed in CAFs under TGFβ regulation in lots of mesenchymal-derived tumors and solid tumors. LRRC15^+^CAF have been found to hamper the function of CD8^+^ T cells, and specific consumption of LRRC15^+^CAF using Lrrc15-diphtheria toxin receptor inhibited tumor growth and increased ICB response [294]. Furthermore, the anti-LRRC15 antibody ABBV-085 has shown efficacious in tumor regression, and then the combination therapy expanded the therapeutic benefits [295, 296]. To date, ABBV-085 has been investigated in a phase I clinical trial (NCT02565758) which enrolled 85 patients with advanced solid tumors to evaluate the safety and pharmacokinetics of ABBV-085 and determine the dose. However, no results have been posted. More importantly, a recent scRNA-seq analysis of fibroblasts from normal pancreas and PDAC provided insights into fibroblast evolution during tumor progression, identifying LRRC15^+^ CAFs of prognostic significance in immunotherapy clinical trials [97]. SLC7A11 (xCT) is a cystine transporter whose therapeutic potential has been established in PDAC. A study found that SLC7A11 abrogation tremendously decreased tumor growth and CAF activation in vitro and in vivo, making targeting SLC7A11 treatment in PDAC-derived CAF a potential therapy [297, 298]. Another glutamatergic pre-synaptic protein Netrin G1(NetG1), was also overexpressed in CAF, associated with CAF metabolism and immunosuppression. Inhibition of this fibroblastic target with neutralizing monoclonal antibody in vivo has been shown to reverse the tumor-promoting function of CAF and change its immunosuppressive function in preclinical mouse models [299]. As mentioned earlier, HSPG2 was expressed by mutant Tp53 to educate CAFs (mt-e-CAF) and contribute significantly to tumor metastasis. The researchers showed that consuming perlecan in combination with chemotherapy prolonged the survival of mice. Given that perlecan expression is predominantly mediated by nuclear factor kappa-B (NFκB) signaling, NFκB inhibitors were proposed as a possible agent to decrease perlecan in pancreatic CAF, and the therapeutic value of perlecan was also discovered in TNBC. Moreover, anti-HSPG drugs such as necuparanib have been reported to lead to MMP1 activity restriction and tissue inhibitor of metalloproteinase 3 (TIMP3) increase in PC patients [129, 130, 300–302]. Nevertheless, PDAC is primarily characterized by fibrous hyperplasia, closely related to PSC and CAFs. Heat shock protein 90 (HSP90) has been shown to play an essential role in tumor and immune system regulation. Despite the crosstalk between PSC/CAF and HSP90 being still unclear, HSP90 inhibition by XL888 can attenuate tumor growth in vitro and enhance the efficacy of anti-PD1 therapy in vivo, which may guide the subsequent direction of research [303, 304].

#### Reprogramming CAF and targeting CAF-associated signaling pathways

In addition to depleting CAFs via their biomarkers or related proteins, another strategy to target CAFs is to weaken or eliminate their pro-tumorigenic functions. In this scenario, some studies have reprogrammed activated CAFs to quiescence, even converting their tumor-promoting phenotypes to tumor-suppressing phenotypes. Since vitamin A deficiency in patients with PDAC leads to PSC activation, restoring retinol stores in PSC by ATRA may reset PSC into a quiescent phenotype. Such reversion of PSCs facilitated apoptosis of surrounding PC cells and decreased proliferation via inhibiting Wnt-β-Catenin Signaling [305]. The combination of ATRA and gemcitabine was found effective in restraining PDAC progression in mouse models. This combination was mediated through a range of signaling cascades (Wnt, hedgehog, retinoid, and FGF) in cancer and stellate cells [306]. Moreover, a phase Ib clinical trial for patients with advanced, unresectable PDAC demonstrated that re-purposing ATRA as a stromal-targeting agent with gemcitabine-nab-paclitaxel is safe and tolerable [307]. These outcomes offer enormous opportunities for ATRA to act as potential drugs to treat PC. Besides, in another study, pharmacologically stimulating vitamin D receptors (VDR, a master genomic suppressor of activated PSCs) by VDR ligand calcipotriol successfully inactivated PSCs. VDR ligand-induced stromal reprogramming reduced cancer-associated fibrosis and inflammation and enhanced the efficacy of a co-administered chemotoxic agent, gemcitabine [308]. Of note, pharmacological reprogramming of CAFs has only been achieved in PDAC contexts. More research is needed to determine whether the reprogramming strategy works in other cancer types.

Similar to previous descriptions of BC, drugs targeting the CXCL12-CXCR4 axis also played a non-negligible role in PC treatment. As AMD3100 synergistic anti-PD1 therapy has been shown to attenuate the number of cancer cells in mouse models significantly, a phase 2 trial (NCT04177810) was conducted in patients with metastatic PC to investigate the combination efficacy of AMD3100 and cemiplimab. The study enrolled 25 participants who will be administered Cemiplimab intravenously (350 mg) on day 1 in a 21-day cycle and AMD3100 at a dose of 80mcg/kg/hr as a continuous intravenous infusion of the first 7 days of each cycle, no results posted [309]. Importantly, AMD3100 was able to deal with immune suppression. Continuous infusion of AMD3100 induced intratumoral T lymphocyte aggregation in patients. It unexpectedly activated the B-cell response, and most patients showed an enhanced T, B cell response after only one week [257, 310]. Based on the immunosuppressive microenvironment of PC, some patients have little or no response to immunotherapy. Of note, a mutual feature of decreased expression of the chemokine CXCL12 was observed in a subpopulation of resistant cells in KPC mice models. Additionally, the use of AMD3100 changed the phenomenon that adding CXCL12 reversed the resistant phenotype, indicating that lack of CXCR12 might cause drug resistance in human PC. However, a phase 1/2 study (NCT02472977) of the safety and therapeutic efficacy of anti-PD-1 antibody nivolumab and anti-CXCR4 monoclonal antibody ulocuplumab was terminated due to lack of efficacy in the short-term acute phase [311]. These pieces of evidence suggested altogether that the sophisticated mechanisms of the CXCL12-CXCR4 axis are still not fully learned and require more in-depth study and dissection. Notable, polymeric AMD3100 (PAMD) was reported to enhance drug delivery of siRNA nanoparticles in PDAC. For instance, PAMD modified with hydrophobic tetrafluoro-p-toluic acid (TFTA) and conjugation of α-tocopherol (TOC) to PAMD indicated higher cellular uptake and tumor accumulation [312, 313]. Another phase 2 clinical trial (NCT02826486) enrolled 80 participants with metastatic pancreatic adenocarcinoma has been designed to investigate the combination of CXCR4 inhibitor BL-8040 (motixafortide), anti-PD1 mAb pembrolizumab, and chemotherapy of onivyde. Although the outcome remains unknown, it is expected to increase patients’ objective response rate (ORR). In addition, a phase II clinical trial of triple therapy, including mopisafortide, pembrolizumab, and NAPOLI-1 regimens (nanoliposomal irinotecan, fluorouracil, and calcium folinate), is safe, effective, and tolerable [314, 315]. A phase II study (NCT04543071) with BL-8040, cemiplimab, and combination chemotherapy (Gemcitabine and Nab-Paclitaxel) in pancreas adenocarcinoma is now recruiting. What’s more? Unlike AMD3100, which was found to affect heart rhythm and cause hypotension in a clinical trial (NCT01280955), MSX-122, another small non-peptide molecule, exhibited fewer side effects than AMD3100. Still, the reason why the MSX-122 clinical trial was discontinued remains unknown (NCT00591682) [316]. Based on the current findings, STAT3 signaling pertains to PDAC drug resistance. CAF-derived circFARP1 synergistically increases the expression and secretion of leukemia inhibitory factor (LIF) by inhibiting CAV1 degradation and acting as a miR-660-3p sponge, activating the STAT3 pathway in PDAC cells, leading to gemcitabine resistance [317]. Moreover, dual inhibition of MEK and STAT3 pathway with specific inhibitors showed reduced stromal inflammation and enrichment of mesenchymal stem cell-like CAF phenotypes, alleviating PDAC immunotherapy resistance.

The hedgehog (Hh) signaling pathway is instrumental in embryonic development and tissue patterning. Usually, the Hh signaling pathway in adults is almost entirely silent in tissues, and abnormal activation of the Hh signaling pathway can lead to carcinogenesis. Constitutive activation of the Hh signaling pathway is explicitly associated with cancer development and progression of various solid malignancies, such as basal cell carcinoma (BCC), medulloblastoma (MB), PC, PCa, etc. [318, 319]. SHH pathway was demonstrated to be directly or indirectly involved in tumor angiogenesis, and activation of Hh signaling in CAF can upregulate the expression of CXCR4 and IGF1R in TME. Again, it also triggered CAF Gli1 upregulation and impacted the expression of transcription factor snails in PC cells through paracrine action, enhancing EMT in PC cells [320–322]. Therefore, inhibition of the Hh signaling pathway has emerged as an attractive and promising cancer therapeutic strategy. The Hh signaling pathway can be divided into two different pathways: canonical and noncanonical. In the canonical pathway, cancer-derived sonic hedgehog (SHH) is an activating ligand for transmembrane protein smoothened (SMO), which is present on neighboring CAFs and promotes ECM production [12]. As an essential molecule, SMO is involved in cascade and has become the primary target of Hh signaling pathway inhibitors [323]. So far, few SMO inhibitor has been approved by FDA for oncotherapy. Vismodegib (GDC-0449) is the first FDA-approved SMO inhibitor for treating advanced and metastatic BCC. Sonidegib (LED-225), another potent SMO inhibitor, received FDA approval in 2015 as a new treatment for locally advanced or metastatic basal cell carcinoma. Although CAF-associated SHH signaling is one of the main pathways of the stromal proliferation of PDAC, clinical trials on SHH pathway inhibitors are not progressing well. In 2014, the deletion of SHH in mouse models and the pharmacological reduction of SHH pathways using vismodegib was pointed out by scientists not only did not have the desired tumor suppressive effect but, in some contexts, accelerated the tumor process because it reduced the stromal desmoplasia [12, 197]. The SMO inhibitor Sonidegib has been reported to alter the proportion of CAF subsets by increasing the number of iCAFs and decreasing the number of myCAFs, thereby attenuating CTL aggregation, which is aligned with increased immunosuppression and may lead to worse long-term consequences [324]. Currently, major drugs targeting the SHH pathway can be classified into SMO inhibitors, HHAT inhibitors, anti-HH mAbs, and GLI inhibitors. However, few of them were demonstrated to alleviate PDAC [325–327]. Despite all these frustrating facts, studies still prove the anti-tumor effects of SHH inhibitors in PDAC. RU-SKI 43, an HHAT inhibitor, was reported to hinder PC cell growth via smoothing-independent non-canonical signaling and could potentially treat leiomyosarcoma, according to Ph, S., and colleagues [328, 329]. Moreover, combination therapies have exhibited some promising results. In PC mouse models, simultaneous targeting of CXCR4 and hedgehog pathways with AMD3100 and vismodegib can improve the therapeutic efficacy of gemcitabine. A more significant reduction in Ki67-positive cells was observed in triple-treatment mice compared with gemcitabine alone, indicating that tumor growth was almost completely inhibited, which solved the problem of chemotherapy resistance to some extent [330]. Another combination of PEG-Gem-cisPt-MSNs and synthetic consisting of SHH inhibitor, cyclopamine (CyP), and mesoporous silica nanoparticles (MSN) have been corroborated in vivo evaluation to enhance the efficiency of drug delivery to tumor cells and reduce tumor mass [331]. MATRIX is a phase I/II study (NCT02358161) showing the safety and efficacy of combination therapy including Sonidegib, gemcitabine, and nab-paclitaxel in participants with metastatic PC. The scientists identified that the maximum tolerated dose (MTD) of LDE225 is 200 mg once daily, and co-administered with gemcitabine 1000 mg / m^2^ and nab-paclitaxel 125 mg / m^2^. They further found that 13% of patients had a partial response (PR), 58% of patients had stable disease (SD), and 29% of patients showed progressive disease (PD). The median OS was 6 months. Moreover, six treatment-related grade 4 adverse events (AEs) and three grade 5 AEs were observed in phase 2 [332]. Moreover, CXCR2 signaling was found to be involved in iCAF formation and CAF to myCAF conversion. Studies previously done by researchers suggested that blockade of the CXCR2 axis decreased tumor angiogenesis and PDAC invasion. Combined inhibition of CXCR2 and CSF1R can reduce granulocyte intratumor infiltration and exhibit strong anti-tumor efficacy [128, 333–335]. Saxena, S. and colleagues revealed that diverse CXCR2 ligands can potentially become diagnostic markers for PC patients [336].

#### Targeting CAF-mediated immunosuppression

In PDAC, CTL and NK T cells are selectively excluded, allowing cancer cells to evade immune surveillance. This makes increasing CTL aggregation or combating the immunosuppressive TME a therapeutic strategy. Based on the use of an anti-mesothelin monoclonal antibody (MSLN Ab) can inhibit the transformation of mesothelial cells into fibroblasts in mouse models of liver fibrosis, as well as the transformation relationship between apCAF and mesothelial cells, researchers found that using MSLN Ab can diminish mesothelial cell to apCAF transition *in vitro* and *in vivo*. Furthermore, this treatment drastically lowered tumor weight and reduced iTreg abundance while increasing the percentage of CD8^+^ T cells in the meantime [183, 337]. Hypoxia-inducible factors (HIFs), first discovered by Semenza and Wang in 1992, are the predominant regulators of the response to hypoxia, and HIF-2α was found to be indispensable for Treg functions. A recent study observed that deleting CAF-HIF2 in mice hindered the intratumoral recruitment of M2 macrophages and Tregs, which were closely associated with immune suppression. Furthermore, after utilizing PT2399, a HIF-2α antagonist used previously to target renal cell carcinoma, tumor responses to immunotherapy were enhanced [338–341]. Proline isomerase Pin1 was found overexpressed both in cancer cells and CAFs, which was correlated with the immunosuppressive TME and poor OS, and Pin1 inhibitor can increase the infiltration of CD8^+^ CTLs, reduce immunosuppressive cells, and may expand the benefits of chemotherapy in GDA and KPC mice [342]. Notably, Liu, J. and colleagues designed a DNA-barcoded micellular system (DMS) functionalized with CAF-targeting anti-FAP-α antibodies (antiCAFs-DMS) that could deliver AG17724, which was a Pin1 inhibitor directionally to CAFs. Furthermore, DNA aptamer was introduced to induce CD8^+^ T cell infiltration, forming the anti-CAFs-DMS-AptT. Anti-CAFs-DMS-AptT was demonstrated to eliminate established tumors and alter or regulate TME in subcutaneous and orthotopic PC models [343].

#### Targeting tumor stroma

**Fig. 4:**
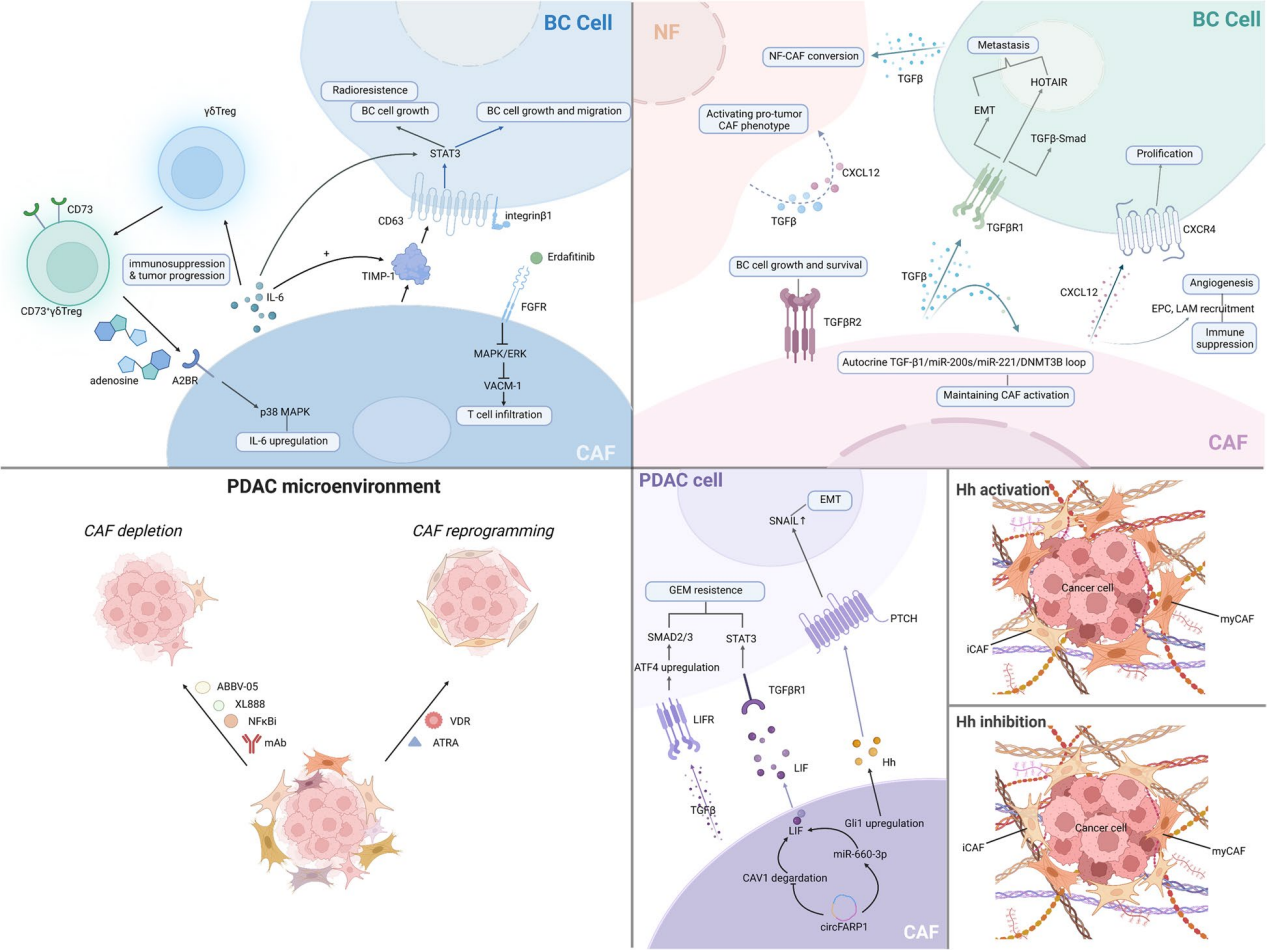
Schematic diagram of the interaction of CAF with cells in BC and PDAC TME. The IL6-adenosine loop potentiates immunosuppression and BC progression, and the TIMP-1/CD63/integrin β1/STAT3 loop is associated with BC cell growth. Erdafitinib promotes T lymphocyte infiltration via inhibiting MARK/ERK signaling. Moreover, CAF-secreted TGF-β1 activates the transcription of HOTAIR to promote BC cell metastasis; the autocrine TGF-β1/miR-200 s/miR-221/DNMT3B loop maintains CAF activity and promotes BC progression. CAF-secreted CXCL12 favors BC cell proliferation and EPC, LAM recruitment. In PDAC, CAF reduction can be achieved by depleting or reprogramming CAF. CAF-derived circFARP1 and TGF-β can both lead to gemcitabine resistance in PDAC cells, and CAF-secreted Hh promotes EMT via upregulating SNAIL transcription. In the end, Hh inhibition changes the proportion of CAF phenotypes in PDAC TME. By Biorender

Hyaluronan (HA) is a glycosaminoglycan (GAG), and a significant component of normal ECM overexpressed in several solid malignancies [344]. HA and collagen collaborate to promote the accumulation of substantial stress (pressure from solid tissue components), which compresses tumor blood vessels [345, 346]. Losartan has been shown to reduce TGF-β-mediated activation of CAFs, reduce the development of desmoplastic tissue components like hyaluronan and collagen produced by CAFs, and increase drug delivery and the effectiveness of immunotherapy [347]. Losartan and other traditional chemotherapy drugs in treating PC are undergoing clinical trials. In a phase II clinical trial that included patients with local advanced PC, neoadjuvant therapy with FOLFIRINOX combined with losartan and chemoradiotherapy demonstrated a high R0 resection rate and prolonged total survival rates [348]. Moreover, enzymic depletion of HA via pegylated recombinant human hyaluronidase (PEGPH20) has attracted researchers’ attention. Shreds of evidence from preclinical studies in PDA mice models demonstrated that PEGPH-mediated HA depletion could decrease interstitial fluid pressure (IFP), improve vascular perfusion, and elevate chemotherapeutic delivery [345, 349]. A phase Ib clinical trial (NCT01453153) enrolled 28 participants with stage IV previously untreated PDAC demonstrated that PEGPH20, combined with gemcitabine, has shown desirable tolerance and may have therapeutic benefits in patients with advanced PC. The PR rate after treatment of PEGPH20 was 35.7%, which was higher than the ORR rate using gemcitabine alone (7–13%), and the treatment-emergent AEs was 96.4% [350]. Additional clinical trials, including phase II and phase III, were conducted to investigate the value of PEGPH20 plus standard chemotherapy regimens (FOLFIRINOX and AG) in treating metastatic PC [351–353]. However, an early phase 1 study of PEGPH20 and Avelumab to treat chemotherapy-resistant PC was terminated for an unknown reason (NCT03481920). Some abovementioned mechanisms are summarized in Fig. 4.

#### Lung cancer

Only about 20 percent of lung cancer (LC) patients survive for five years. LC was the deadliest in 2019, claiming approximately 2 million lives worldwide. The most common LC was NSCLC, known for its uncomplicated metastasis and drug resistance, but the mechanism is still unclear [354]. But in recent years, with the increase in awareness of CAF, strategies targeting CAF have proven effective in LC treatment.

#### Targeting CAFs via biomarkers

The CAR-T strategy has made noticeable progress in LC treatment. According to some studies, FAP can be genetically engineered to become a viable target for CAR. In preclinical studies, FAP-specific CARs were developed to redirect T cells to FAP-positive CAFs. These T cells can form a specific immune attack against FAP^+^ CAFs, with concomitant antitumor efficacy and no apparent signs of toxicity [355, 356]. Prior studies have shown that FAP-CAR T cell therapy in human LC xenografts and homologous mouse PC models can reduce tumor vascular density, restrain desmoplasia, and grow native PC [357]. Lately, a phase I clinical trial (NCT01722149) enrolled four patients with metastatic pleural mesothelioma (MPM) using FAP targeting CAR T-cells (CART-FAP) have been reported safe, with major SAEs including upper respiratory infection and thromboembolic event. Intra-pleural injection of single CART-FAP in MPM patients is safe and increases proinflammatory cytokines levels in patients’ sera. However, due to the small number of patients enrolled, the impact of this treatment on patient outcomes could not be evaluated and entailed further investigation [358]. More recently, the combination of Nectin4-targeted CAR-T (Nectin4-7.19 CAR-T) and FAP-targeted CAR-T (FAP-12 CAR-T) cells was demonstrated by Li, F., and colleagues to exterminate lung metastasis in the NSG mouse model. Furthermore, the radiolabeled FAP inhibitors applied to PET imaging can potentially monitor therapeutic response to FAP-targeted CAR T-cell therapy, reducing the limitations of CAR T-cell therapy. In addition, the therapeutic effect of CART is also gradually established in HCC and glioblastoma [359–363]. Unfortunately, it has been observed that using FAP-targeted CAR T cells to target subcutaneous tumors in mice led to deadly myelotoxicity and cachexia [224].

#### Targeting the signaling pathways of CAFs

The TGF-β signaling pathway promotes EMT through the secretion of IL-6 in NSCLC. It induces tumor tissue remodeling by transforming entities to acinar in LC cells, affecting the histological pattern of lung adenocarcinoma (LUAD) [364, 365]. CUDC-907 is a dual inhibitor of the HDAC and PI3K/AKT pathways that inhibited the proliferation and differentiation of CAF, which was induced by TGF-β1, as well as the collagen expression. In a phase 1b/2 study (NCT02423343), scientists enrolled 41 participants who suffered from advanced solid tumors and recurrent NSCLC or HCC. In phase 1b, patients were divided into four cohorts receiving Galunisertib orally at the dose of 50 mg once daily, 50 mg twice daily, 80 mg twice daily, and 150 mg twice daily on Day 1 through Day 14 of each 4-week cycle combined with 3 mg/kg nivolumab given intravenously until discontinuation criteria are met. Researchers identified that the MTD of Galunisertib was 300 mg. In phase 2, patients with NSCLC or HCC were administered Galunisertib 150 mg twice daily on Day 1 through Day 14 of each 4-week cycle combined with 3 mg/kg nivolumab given intravenously. The results showed that when administered with Galunisertib, no patients were found to have anti-Nivolumab antibodies. The median PFS was 5.26 months, and the ORR was 24% in patients with NSCLC. The immunogenic effects of radiotherapy (RT) may be counteracted by avoidance mechanisms in TME, including induction of angiogenic factor secretion and CAF activation. At the same time, BA can reverse radiotherapy-induced CAF activation and fibrosis. Altogether, Y, L., and colleagues suggested that BA + RT (BART) combinations potentially eradicated drug-resistant tumors while preserving normal tissue [366]. Moreover, Shi, X., and colleagues revealed that TGF-β inhibitor LY2109761 decreased the expansion of squamous cell carcinoma (SCC) CAFs in the lung in vivo, and another TGF-β inhibitor, LY2157299, can inhibit the formation and invasion of CSC-CAFs co-cultured spheres in vitro [367].

P62 has been found to be associated with lung adenocarcinoma progression. Inhibition of autophagy with hydroxychloroquine (HCQ) reduced CAF activation and TGFβ production, thereby hindering tumor growth. Mechanistically, p62-induced autophagy upregulates the expression of nuclear factor erythroid 2 correlated factors 2 (Nrf2) and activated transcription factor 6 (ATF6) to promote CAF activation and pharmacological inhibition of the Nrf2-ATF6 pathway can completely block CAF activation [368]. In addition, CAF-derived SDF-1 induced the EMT of LUAD cells via CXCR4/β-catenin/ PPARδ signaling. Thus, using the β-catenin inhibitor XAV-939 and PPARδ inhibitor GSK3787 to target the CXCR4 β-catenin/ PPARδ cascade attenuated EMT, which might serve as a potential therapy for LC treatment [369]. Moreover, the third-generation EGFR inhibitor osimertinib treats tyrosine kinase inhibitor (TKI)-resistant NSCLC, and most patients eventually become osimertinib-resistant. Studies have shown that the MEK inhibitor trametinib can eliminate the high expression of FAP and excessive secretion of IL-6 in osimertinib-resistant cells by inhibiting the MEK / ERK / miR-21 axis. In the xenograft model, osimertinib and trametinib combination therapy had a significant growth-inhibiting effect on osimertinib-resistant NSCLC tumors [370]. Li, H. et al*.* also unraveled that the intracellular accumulation of reactive oxygen species (ROS) can activate the STAT3 pathway, allowing senescent fibroblasts to exhibit CAF signatures, thereby expediting the migration of H1299 and A549 LC cells [371].

#### Other targets

Metabolic reprogramming is a crucial feature of cancer that allows cancer cells to survive and proliferate wantonly. Recent studies have shown that cancer cells exhibited high glutamine uptake in TME [372, 373]. A CAF-specific lncRNA, LINC01614, enhanced glutamine metabolism in LUAD cells by promoting NF-κB activation and upregulating the expression of glutamine transporters SLC38A2 and SLC7A5. Furthermore, the pro-inflammatory factors secreted by cancer cells, such as IL-6 and CXCL10, can upregulate the expression of LINC01614 through a feedforward loop. Liu, T., and colleagues stated that LINC01614 was associated with poor prognosis in patients and that deleting LINC01614 reduced metastasis in NCG mice and zebrafish cancer models, suggesting that targeting particular lncRNA can attenuate glutamine utilization in cancer cells, potentially delaying cancer progression [374].

TAM was reported to promote CAF formation through MMT, and Smad3 was one of the critical regulators in this process. Macrophage‐lineage CAF and tumor growth were significantly impaired in Lewis lung carcinoma (LCC) mice models after utilizing the SMAD3 inhibitor, SIS3 [72]. In addition, because SIS3 was identified to promote NK cell cytotoxicity by improving Smad3-mediated inhibition of Ndrg1 transcription, it also made it one of the potential therapeutic strategies for LC [375]. Besides, research concentrating on reprogramming CAFs has pointed out that when CAFs were exposed to apoptotic cancer cells, apoptotic cancer cells reprogramed CAF through the Notch1-WISP-1 signaling pathway, inhibiting cancer invasion and metastasis. This effect was also demonstrated in mice, where injection of apoptotic 344SQ cells (ApoSQ) inhibited CAF activation, as evidenced by reduced mRNA levels of CAF cytokines [376].

#### Colorectal cancer

CRC was the fourth deadliest cancer in the world in 2019. Because its symptoms are usually insidious in the early stages, how to recognize CRC early is an overarching problem to overcome. Currently, the treatment methods for CRC mainly include endoscopic and surgical local resection, palliative chemotherapy, targeted therapy, and immunotherapy [377]. However, research and drugs targeting CAF are still being established in CRC, unlike other cancers. Several genes associated with CAF are possible therapeutic targets, including COL3A1, JAM3, AEBP1, WNT2, and WNT54 [378, 379]. Research on the origins of CAF recently revealed that many ACTA2 CAFs are derived from the proliferation of intestinal pericryptal leptin receptor (Lepr) cells expressing melanoma cell adhesion molecules (MCAM) through lineage tracing. In mouse models, matrix MCAM knockout attenuated colorectal tumoroid growth injected in situ. Therefore, preventing the differentiation of leprosy spectrum CAF or inhibiting the activity of MCAM might be an effective treatment for CRC [380]. Below, we will elaborate on other approaches to target CAF.

#### Targeting signaling pathways

Targeting TGF-β pathway strategy is also described in CRC. CAF-derived exosomes boosted stemness of CRC cells via TGF-β signaling, thereby improving radiation resistance, and this effect was attenuated after the use of neutralizing antibodies [381]. Yang, M., and colleagues revealed that the overexpression of fibronectin leucine-rich transmembrane protein 3 (FLRT3) can restrain EMT, and FLRT3 downregulation is associated with poor prognosis. CAF reduced FLRT3 expression by activating the TGF-β/SMAD4 signaling pathway and enhanced CRC aggressiveness. TGF-β inhibitor LY2109761 can attenuate this effect, reducing the amount of Treg in TME [382]. Besides, resveratrol (RES) can also suppress EMT through TGF-β1/Smads signaling in CRC [383]. Intriguingly, resveratrol-loaded liposome (L-RES) reduced α-SMA and IL-6 levels in activated fibroblasts and disrupted crosstalk between CRC cells and CAF to inhibit the function of CAF [384]. The anti-tumor role of RES was also observed in BC, LC, and PCa [385–387]. According to Naktubtim, C. and colleagues, YAP regulates CAF transformation associated with F-actin rearrangement, thereby promoting CRC cells' proliferation, migration, invasion, and angiogenesis. The YAP inhibitor verteporfin (VP) can reverse the above reaction. More recently, the IGF2-IGF1R-YAP1 axis was demonstrated to be a prognostic biomarker and therapeutic target for CRC. The expression of IGF2 in CAF was upregulated, while IGF1R was mainly expressed by cancer cells. IGF2 can cause YAP1 to accumulate within the nucleus. However, the cascade activation mediated by IGF2 was dispelled by IGF1R depletion and an IGF1R inhibitor, picropodophyllin (AXL1717). Moreover, the AXL1717 and VP combination therapy showed greater anti-tumor efficacy than PPP alone [388, 389]. γ-mangostin (γ-MG), a critical active substance isolated from mangosteen, inhibited the GSK3/β-catenin/CDK6 pathway associated with CRC stemness. Using gMG in xenograft mouse models inhibited tumor growth and overcame CAF-induced 5-fluorouracil resistance [390]. It is reported that CAF-secreted IL-6/IL-11 can activate STAT3 signaling, further facilitating CRC progression. Correspondingly, inhibiting the activation of the STAT3 pathway in COL1^+^ CAFs can impair CRC development in the AOM/DSS model [391, 392]. Although therapeutic trials using STAT3 inhibitors have demonstrated significant results in certain oncological diseases, a randomized phase 3 trial showed that OS in the patient cohort was not expected when the STAT3 inhibitor napabucasin was used for refractory metastatic CRC [393].

#### Targeting CAF-derived exosomes

The exosomal LncRNA LINC00659, originating from CAF, has been shown to promote the proliferation and migration of CRC cells by activating ANXA2 and downregulating the expression of miR-342-3p. In addition, exosomes miR-146a-5p and miR-155-5p were found to have increased expression in CXCR7-overexpressed CRC cells. These two exosomes can be absorbed by CAF, which is conducive to the activation of CAF. Functional studies have shown that activated CAFs highly express inflammatory factors such as IL-6 and CXCL12 to promote the invasion and metastasis of CRC cells. Significantly, miR-146a-5p and miR-155-5p activate CAFs to promote tumor formation and lung metastasis of CRC in vivo in tumor xenograft models [394, 395]. Other CAF-derived exosomes such as WEE2-AS1 and circEIF3K have also been reported to potentiate CRC progression via inhibiting the Hippo signaling pathway and activating the miR-214/PD-L1 axis, respectively [396, 397]. Furthermore, certain exosomes contributed to CRC angiogenesis, chemoresistance, and radioresistance [398–400]. Taken together, targeting exosome therapy was suggested to become a promising strategy for CRC treatment. Moreover, CAF-derived lncRNA (CAFDL) can predict the prognosis of LUAD, BC, thyroid cancer, and many other cancers. CAFDL can be used as a risk stratification tool to predict the clinical outcome of CRC [401]. However, specific drugs targeting exosomes have rarely been reported, so it is urgent to study new agents and verify their clinical safety and feasibility.

#### Targeting TME and metabolism

Shen, W., and colleagues designed a nanoemulsion combining chemotherapy and gene therapy to simultaneously deliver doxorubicin and small interfering RNAs targeting hepatocyte growth factor (HGF) to reduce ECM deposition, induce CAF apoptosis, and reduce tumor metastasis. The chemotherapy resistance was ameliorated somewhat, and the tumor progression was inhibited [402]. Another tyrosine kinase inhibitor, NT157, can pharmacologically inhibit IGF-1R and STAT3 signaling. It can target CRC by simultaneously influencing tumor-associated cells and their supportive microenvironment, such as inhibiting the secretion of pro-tumor cytokines [403]. As mentioned in LC, CAF can also impact the metabolism of CRC. CAF reprograms CRC metabolism by stimulating glycolysis, oxidizing arms of the pentose phosphate pathway (PPP), and inhibiting the tricarboxylic acid cycle. The researchers found that knocking out hexokinase and glucose-6-phosphatase in CAF-treated CRC cells slowed tumor growth. They also proposed that using inhibitors of these two enzymes, including metformin and polydatin, as well as emerging bispecific antibodies and proteolytic targeted mosaicism (PROTACs), could be a potential therapeutic approach [404]. Additionally, MMP14-expressing CAFs were found to be associated with CRC progression, and patients with high MMP14 CAF/CAF ratios exerted adverse outcomes, which may have prognostic value [405].

#### Prostate cancer

The incidence of PCa ranks second in the world among male malignancies. In 2022, the incidence of prostate cancer in the United States has surpassed LC, becoming the most severe malignant tumor that endangers men's health. The treatment methods for PCa mainly include endocrine hormone therapy, radiotherapy, chemotherapy, surgical treatment, emerging immunotherapy, etc. Among them, castration therapy is an imperative treatment, but cancer cells will inevitably become resistant to castration therapy. Studies have reported that CAF has a promoting effect on castration resistance. Coupled with the previously described CAF functions, targeting CAF and its related activities has become a promising treatment. Moreover, some CAF gene signatures such as ACPP, THBS2, TMEM132A, and ZNF467 were established to estimate the survival of PCa patients undergoing radiotherapy [406].

#### Targeting CAFs specific biomarkers and signaling pathways

Targeting FAP strategies have also been proposed and applied in PCa. A nanosystem delivering FAP antibodies can inactivate CAF by downregulating the expression of CXCL12 to modulate the tumor microenvironment of PCa [407]. ^68^Ga-FAPI was highly uptaken by PCa cells, so it is used for diagnosis in PET/CT imaging [408, 409]. Moreover, Kakarla, M. and colleagues unraveled that CAF showed higher expression of Ephrin B1, B2, and B3 ligands (EFNB1, EFNB2, and EFNB3) compared to NF. It has also been mentioned that high levels of EFNB1 and EFNB3 in benign human prostate stromal cell lines can potentiate tumorigenicity in PCa cells and activate Src family kinases (SFKs) in prostate fibroblasts. SFK inhibitor AZD0530 (Saracatinib) decreased the expression of the CAF marker ɑ-SMA and ECM protein TNC in vitro [410]. As for CAF-associated signaling pathways, targeting the CXCL12/CXCR4 axis has also shown novel advances in PCa. For instance, the aforesaid AMD3100 can reduce tumor angiogenesis and decrease the migration of tumor endothelial cells (TEC) in vitro [411]. Alternatively, the inhibition of TGFβ and CXCL12 can overcome the immune suppressive microenvironment. MPSSS, a natural polysaccharide extracted from shiitake mushrooms, disrupted T cell inhibition mediated by CAF via activating TLR4-NF-κB signaling [412]. Notably, the decreased expression of some CAF biomarkers and TGFβ2 caused by silibinin was observed in PC3 tumors, which attenuated NFs to CAFs conversion, suggesting the therapeutic potential of these natural flavonoid lignans. Again, FOXF2 (Forkhead Box F2) in the prostate stroma reduced the iCAF phenotypic ratio, induced its transformation to myCAF, and downregulated CXCL5, thereby reducing immunosuppressive myeloid cells and enhancing T cell cytotoxicity. Further, increasing the content of Foxf2 sensitized PCa to ICB therapy [413]. The gene set variation analysis by Zhai, X., and colleagues indicated that pathways like PPAR, GnRH, and mTOR are significantly correlated with PCa and might become targeted landmarks [414].

#### Targeting CAF-induced castration resistance

Castration therapy is currently one of the mainstream treatments for PCa. Still, castration resistance is also a troublesome problem, and emerging evidence validates that CAF contributes to resistance via diverse mechanisms. When castration-resistant PCa CSC and castration-resistant prostate cancer CAF (CRPCAF) were Adisetiyo, H. and colleagues observed co-cultured, more aggressive tumorigenesis. CRPCAF had a far more powerful ability to support organoid/tumor formation than androgen-dependent PCa CAF in vivo and in vitro, suggesting that CRPCAF can improve the stemmatic and tumorigenic properties of CSCs [415]. Androgen receptor (AR) is normally expressed in prostate epithelial cells, PCa cells, and prostate fibroblasts. It has been reported that AR inactivation upregulated LIM domain only 2 (LMO2) expression in prostate CAF, and paracrine IL-11 and FGF-9 can activate pathways such as STAT3, AKT, etc., which subsequently activated AR in PCa cells, leading to castration resistance. Correspondingly, the use of neutralizing antibodies or pathway blockade exhibited a decrease in AR activation [416]. Research showed that CD105 CAF could mediate neuroendocrine epithelial differentiation and castration resistance in prostate tumors in a paracrine manner [417]. Neuromodulator 1 (NRG1) secreted by CAF can promote tumor cell resistance to antiandrogen therapy by activating HER3. The use of pharmacological inhibition of the NRG1-HER3 pathway with YW538.24.71 (NRG1-neutralizing antibody), AMG888 (HER3-blocking antibody), and neratinib (HER2 kinase inhibitor) significantly inhibited the growth of CWR22Pc tumor, a patient-derived xenograft model. It restored their responsiveness to anti-androgenic therapy [418, 419]. Glucosamine secreted by CAF has increased O-GlcNAcylation in PCa cells, thereby promoting Elk1-mediated HSD3B1 transcription. The expression of the vital enzyme 3β-Hydroxysteroid dehydrogenase-1 (3βHSD1) of the extragonadal androgen synthesis pathway, which HSD3B1 encoded was increased. As a result, the synthesis of androgens in tumors can be upregulated to form castration resistance. Blocking Elk1 inhibited CAF-induced androgen biosynthesis in vivo [420].

#### Targeting metabolism and TME

One of the energy metabolism characteristics of tumor cells was identified as heavy dependence on glycolysis and the production of large amounts of lactic acid, which is deposited to promote tumor progression by multiple mechanisms. In PCa, studies determined that CAF predominantly secretes lactate, and tumor cells store lipids into lipid droplets to support mitochondrial metabolism. Inhibition of BET with I-BET762 (Molibresib) to target histone acetylation interfered with lactate-dependent lipid metabolism and could impede prostate cancer growth and metastasis in mice [421–423]. Furthermore, an open-label phase I trial (NCT01587703) of Molibresib has been conducted in patients with castration-resistant prostate cancer (CRPC), CRC, NSCLC, and other cancers. The study consisted of two parts. Part 1 of the study was a dose escalation establishing the recommended dose for part 2, and part 2 assessed the safety, pharmacokinetics, and pharmacodynamics of the recommended dose. It is indicated in part 1 that oral administration of Molibresib 80 mg once daily was the recommended phase 2 dose. In part 2, the results showed that only one participant with CRPC completed the study. 84 patients (82%) in all cohorts suffered from grade 3/4 AEs, mostly thrombocytopenia (43%) and anemia (21%). Only one confirmed PR was reported in CRPC. In conclusion, treatment with Molibresib 75 mg once daily was tolerable but may require dose interruptions. And the anti-tumor efficacy entailed further investigation. Alternatively, Neuwirt, H. et al*.* revealed that CAF could boost cholesterol and steroid biosynthesis levels in PCa cells by highly upregulating the expression of HMGCS2 and AKR1C3 to promote androgen receptor-targeted therapy resistance in PCa. Notably, the dual inhibition of cholesterol and steroid synthesis with simvastatin and AKR1C3 inhibitors demonstrated significant tumor growth inhibition [424].

In 2017, the overexpression of LOXL2 in PCa tissues was confirmed. Two years later, Nguyen EV and colleagues found through proteomic analysis that proteins related to cell adhesion and extracellular matrix were significantly enriched in CAF, including LOXL2, discoidin domain-containing receptor 2 (DDR2), etc., compared with normal fibroblasts. Of note, using LOXL2 inhibitors D-penicillamine and PXS-S2A can hinder CAF migration and ECM alignment [145, 425]. Besides, LOXL2 knockdown enhanced castration-resistant PCa radiosensitivity in vitro and in vivo conditions and provided insight for solving the resistance of PCa radiotherapy [426]. Additionally, a previous study has shown interactions between M2 macrophages and CAF. On one hand, M2 macrophages promoted EMT conversion; on the other hand, CAF secreted SDF-1 to recruit monocytes and facilitated their differentiation into M2 macrophages. G protein-coupled receptor 30 (GPR30) is an estrogen receptor highly expressed in prostate CAFs. Knocking it out hinders macrophage infiltration and M2 polarization, adversely affecting PCa invasion [427, 428]. Subsequently, BXCL701, a small molecule inhibitor of dipeptidyl peptidase (DPP) that can trigger inflammation within TME and increase immunotherapy responsiveness, was recently studied in a phase 1b/2 study (NCT03910660) that enrolled 95 participants to evaluate the efficacy and safety of BXCL701 oral, monotherapy, and in combination with pembrolizumab (PEMBRO) in patients with mCRPC. The trial was divided into three parts, the first part described above, and the second part divided patients into two cohorts by cancer type: small cell neuroendocrine prostate cancer (SCNC) and adenocarcinoma phenotype to observe the composite response rate of BXCL701 + PEMBRO. Phase 2b of the study will only enroll patients with histological subtypes showing preliminary evidence in Phase 2a, assessing response rates in patients treated with BXCL701 + PEMBRO and BXCL701 monotherapy. However, the study is now active, not recruiting. EXPEL PANC was an ongoing clinical trial (NCT05558982) testing the same drug combination BXCL701 + PEMBRO in patients with metastatic PDAC. The research was expected to improve patients’ progression-free survival at 18 weeks, but the result has not been published.

#### Melanoma

Melanoma, malignant from melanocytes, is the deadliest type of skin cancer, usually triggered by ultraviolet, trauma, etc. Melanoma is expected in the skin but is not limited to the skin, it can also occur in the conjunctiva, oral cavity, etc. FDA-approved immunotherapy like anti-PD-1 therapy and targeted drugs vimofenib have brought hope to melanoma patients, significantly improving their five-year survival rates [429, 430]. Targeting CAF has become a promising treatment strategy for melanoma as well. Studies have pointed out that because stromal fibroblasts and CAFs have higher genetic stability, they are less likely to develop drug resistance than malignant cells.

#### Targeting CAFs-related proteins

To date, conventional therapies against CAF have not been widely reported in melanoma. NRG1, highly expressed in fibroblasts and CAF, is a ligand for ErbB3, and fibroblast-derived NRG1 can attenuate the effect of RAF inhibitors on melanoma cells. Targeting the ErbB3/ErbB2 pathway with neutralizing antibodies seribantumab (MM-121) and pertuzumab counteracted the protective effects of CAF and improved melanoma cell drug responsiveness [431]. Seribantumab has been investigated in 44 patients with advanced solid tumors in a phase 1 clinical trial (NCT00734305). The participants were divided into 6 cohorts and received seribantumab intravenously once a week for a maximum of 47 weeks at the beginning dose of 3.2 mg/kg, and the dose escalated in separate cohorts from 6 mg/kg, 10 mg/kg, 15 mg/kg, 20 mg/kg, to the highest scheduled testing dose at 40 mg/kg one-time loading dose on cycle 1, week 1 followed by 20 mg/kg maintenance doses. ORR is 0 for the 6 cohorts, and the incidence of serious AEs and other AEs was 32.56% and 100%, respectively. The most reported AEs were nausea (46.51%) and fatigue (48.84%). In another phase 2 study (NCT04383210), researchers aimed to assess the ORR of seribantumab in patients with recurrent, locally advanced, or metastatic solid tumors, which harbor the NRG1 gene fusion, but the outcomes remain unknown as well. It is believed that the β-catenin in Wnt/β-catenin signaling might be associated with early melanoma. In a Col1α2-CreER mouse model in which β-catenin was knocked out from dermal fibroblasts, researchers observed a decrease in chemokines and ECM protein production. They proposed that dermal fibroblasts acted as a physical barrier and exerted anti-tumor function before shifting to CAFs. To further substantiate the role of β-catenin in CAF, a study by Zhou, L. and colleagues observed that the β-catenin pathway blockade in CAF magnificently inhibited the proliferation of melanoma in a PTEN-deficient mouse model activated by BRAF and the EMT process of melanoma cells was also suppressed. More recently, BRAF inhibitors induced β-catenin accumulation in CAF, stimulated the secretion of the downstream effector POSTN of β-catenin signaling, and finally activated ERK signaling to allow melanoma cells to continue proliferating in the presence of BRAFi and MEKi, suggesting that explicitly targeting POSTN may be one of the promising options [432–434]. On the contrary, some novel mechanisms have been discovered that may guide the establishment of strategies targeting CAF. According to studies from Papaccio, F., CAF can affect melanoma cell migration and protect melanoma cells from paclitaxel-induced apoptosis [435].

#### Targeting CAF-derived exosomes

Since exosome is a vector for crosstalk between CAFs and melanoma cells, several studies have concentrated on dissecting its inherent mechanism [436, 437]. It was reported that miR-155 derived from melanoma cells could inhibit the expression of tumor suppressor gene SOCS1 in CAF, activate the JAK2/STAT3 signaling pathway, and trigger the transformation of CAF to proangiogenic type. Interestingly, the single inhibition of exosomal miR-155 could not restore the original angiogenic factor levels, indicating that other factors synergistically promoted the proangiogenic conversion of CAF, which remained to be explored [438]. Furthermore, Dror, S. and colleagues isolated 5 microRNAs from mature melanosomes, of which the most abundant miR-211 was shown to be transported from melanocytes to fibroblasts through melanosomes, causing phenotypic changes in co-transplanted mouse models, that is, reprogramming NF to CAF. In terms of mechanism, the response to promote collagen contraction and pro-inflammatory factor secretion was achieved by downregulating the expression of tumor suppressor IGF2R and activating the MAPK signaling pathway. Of note, using the p38 inhibitor SB202190 (FHPI) reduced the secretion of melanosomes and miR-211, and the presence of the ERK (a marker for MAPK signaling activation) inhibitor U0126 also eliminated the effect of miR-211 on NF [439–441]. Notwithstanding, none of these inhibitors have moved into clinical trials yet.

#### Other targets

**Fig. 5:**
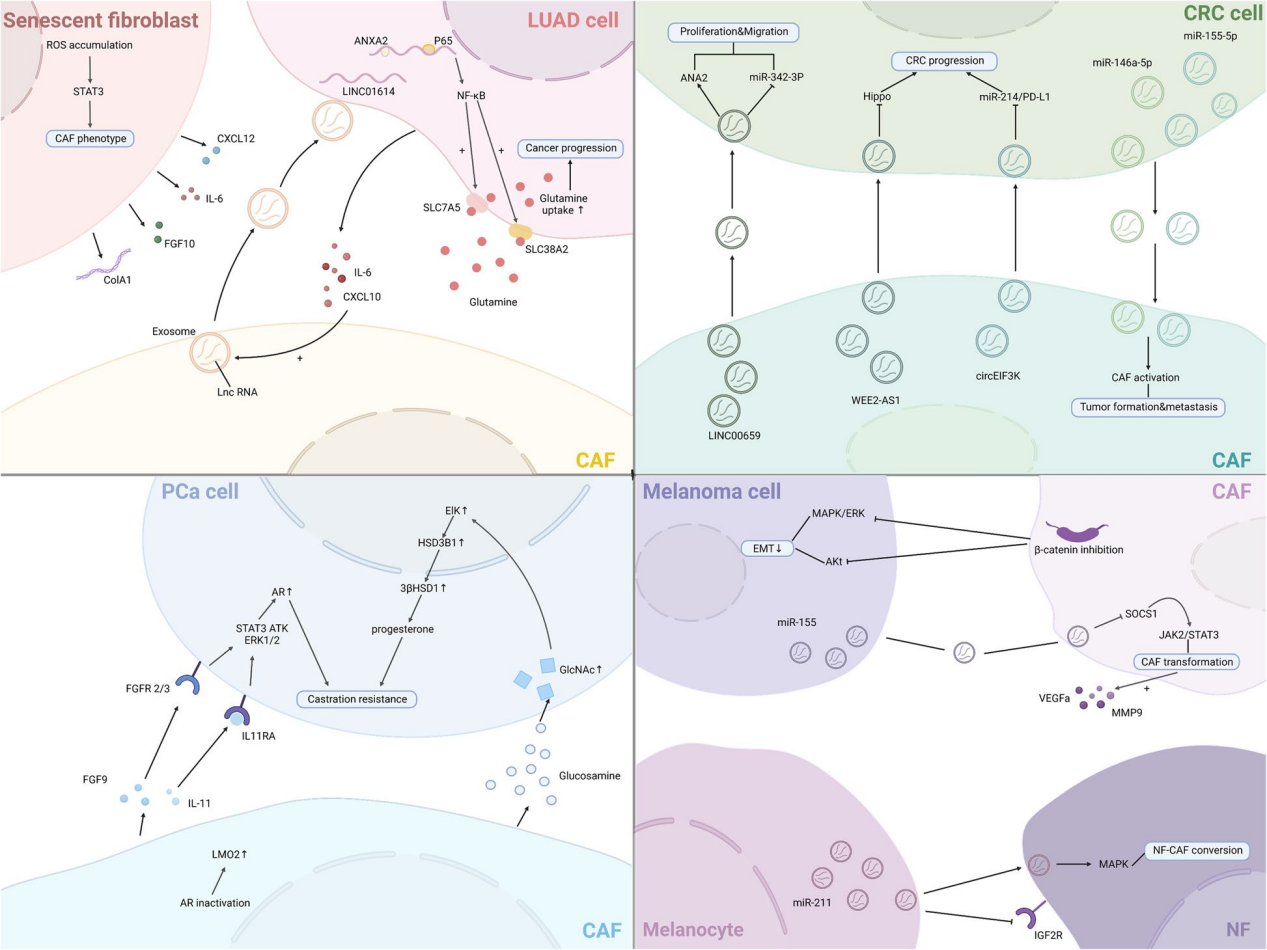
Schematic diagram of the interaction of CAF with cells in LC, CRC, PCa, and Melanoma TME. CAF-secreted exosome LINC01614 activates the NF-κB signaling pathway, thus upregulating the glutamine transporters SLC38A2 and SLC7A5 to promote glutamine uptake. In CRC, the exosomal LINC00659, WEE2-AS1, and circEIF3K from CAF enhance CRC progression through various mechanisms, and the miR-146a-5p and miR-155-5p from CRC cells activate CAF. Moreover, through FGF9, IL-11, and glucosamine secretion, CAF expedites castration resistance in PCa cells. At last, inhibition of β-catenin in CAFs downregulates AKT and MAPK/ERK signaling and blocks EMT in Braf^V600E^; Pten^lox5/lox5^ melanoma. Melanoma cell-secreted miR-155 and melanocyte-secreted miR-211 promote CAF transformation and NF-CAF transition respectively. By Biorender

In addition, a 2021 study found that neutrophils in mouse PC and melanoma models often aggregated in CAF-rich regions, creating extracellular traps (NETs). Specifically, CAF drove tumor-induced NETs (t-NETs) formation through the ROS pathway and amyloid β, and the interaction between CAFs and NETs was reciprocal. The PAD4 inhibitor GSK484 in vivo to inhibit histone citrullination in the ROS pathway in a melanoma mouse model was observed to impair tumor growth. In addition, BACE inhibitors can suppress amyloid β production, thereby hindering tumor development. The blockade of CD11b, a possible receptor of amyloid β on the surface of neutrophils, essentially eliminated NETosis. Eventually, they proposed that the amyloid β-NET axis behaved analogically in human PC and melanoma as in mice, which was associated with poor survival [442]. This study defined a novel mechanism between CAFs and NETs. It provided three potentially valid targets to target the amyloid β-NET axis, paving the way for further investigation in humans. Some abovementioned mechanisms are summarized in Fig. 5.

#### Other cancers

Irrespective of the aforementioned cancers, novel mechanisms, and protocols targeting CAF have also prospered in other cancer types. A cytokine named cardiotrophin-like cytokine factor 1 (CLCF1), derived from CAF, can promote the secretion of CXCL6 and TGF-β in HCC cells in an Akt/ERK1/2-STAT3 pathway-dependent manner. These upregulated chemokines then worked synergistically to expedite stem properties of tumor cells and tumor-associated neutrophil polarization, and TGF-β was also associated with NET formation. This study also demonstrated a positive feedback effect between CXCL6, TGF-β, and CLCF1 by activating the ERK1/2 pathway in CAF. Correspondingly, researchers suggested that selective inhibition of CLCF1/CNTFR (ciliary neurotrophic factor receptor) or ERK1/2 in mouse models precluded crosstalk between CAFs and HCC cells, which might become a potential treatment for HCC patients since up-regulation of CLCF1-CXCL6/ TGF-β cascade in HCC patient samples was pertaining to poor prognosis [443]. A lipid with endogenous anti-inflammatory effects, Resolvin, has been shown to impede tumor progression and increase therapeutic efficacy. In 2019, Sun, L. et al*.* demonstrated that Resolvin D1 (RvD1) impaired CAFs-induced tumor stemness, EMT of HCC cells, and production of cartilage oligomeric matrix protein (COMP), which had an apparent pro-tumor function. Later, RvD1 was shown to influence monocyte recruitment and the activity of tumor-associated neutrophils, reducing the proliferation of HPV-positive cancer cells in mice and humans in vivo and in vitro. Therefore, activating RvD1 or exogenous supplementation may have effective therapeutic outcomes [444–446]. Additionally, the overexpression of SPP1 was found in patients who did not respond to sorafenib, and patients with SPP1‐related gene expression displayed some adverse outcomes. Eun, J. W. and colleagues revealed that CAF-secreted SPP1 could reinforce HCC TKI resistance via activating RAF/MAPK and PI3K/AKT/mTOR signaling and favors EMT as well, indicating that SPP1 blockade may have clinical value [447].

In GC, HSF1-mediated upregulated inhibin subunit beta A (INHBA) and Thbs2 are secreted from CAF via extracellular vesicles, promoting aggressive GC phenotype and progression [448]. More importantly, there is much evidence that CAF is highly correlated with drug resistance in GC. CAF has been demonstrated to confer 5-fluorouracil (5-FU) resistance to GC cells by expressing neuropilin 2 (NRP2), eventually activating the Hippo pathway and alternatively activating JAK/STAT3 in cancer cells via secreting cytokines. Ham, I.-H. and colleagues have claimed that curcumin can inhibit the JAK2/STAT3 pathway activated in GC cells. In xenograft models, curcumin synergistically inhibits the growth of GC with 5-FU, suggesting that this natural substance extracted from ginger has a prominent anti-tumor ability [449, 450]. As mentioned earlier, the stiffened ECM induced by CAF also favored chemoresistance. According to Lu, Y., the overexpression of calponin1 (CNN1) in CAF enhanced the contraction of CAF, leading to stromal sclerosis, and the hardened stroma acted on GC cells, thereby activating YAP signaling and causing chemotherapy resistance [451]. Besides, it has been suggested that the redundant matrix was associated with immunosuppression. A designed drug combination comprising dual PDGFRα/β suppression by regorafenib and anti-PD-1 therapy exhibited good efficacy in treating fibrotic tumors since blocking PDGFRα/β can invert the immunosuppression in murine GAN-KP tumors. Multikinase inhibitor regorafenib itself was demonstrated to decrease CAF-secreted chemokines, thus modulating TME to a less immunosuppressive state [452]. A phase 2 study (NCT01913639) enrolled 39 participants with unresectable or metastatic esophagogastric cancer intended to evaluate the effects and safety of regorafenib with chemotherapy regime (FOLFOX) including 5-Fluorouraci, Leucovorin, and Oxaliplatin. The results showed that 53% of the participants showed PFS, 54% of the participants were with CR or PR, and the median OS was 14.2 months. 17 patients (43.59%) suffered from serious AEs such as abdominal pain (23.08%) and dyspnea (10.26%), and 36 patients (92.31%) suffered from other AEs, mostly fatigue (79.49%), anemia (71.79%), nausea (58.97%), and peripheral sensory neuropathy (69.23%). Another phase 3 study (NCT04879368) evaluating the effectiveness of regorafenib and nivolumab combination compared with the standard chemotherapy (Docetaxel, Paclitaxel, Irinotecan, and Trifluridine/Tipracil) in prolonging the OS in a broad group of 450 participants with advanced gastro-oesophageal cancer (AGOC) is now recruiting. In a CAF-conditioned medium (CAF-CM), CAF-secreted midkine (MK) can activate PI3K/AKT signaling via upregulating lncRNA ST7-AS1 expression in GC cell, ultimately enhancing EMT and cisplatin resistance. Similar results that MK affected xenograft tumor growth and chemotherapy resistance via ST7-AS1/AKT/EMT axis were verified in vivo [453]. Thus, targeting the interactions between CAF and drug resistance might become a therapeutic option.

Bladder cancer is the most common malignancy of the urinary system. ScRNA-seq defined an interferon-regulated subpopulation of bladder cancer CAF, featured by overexpression of the urea transporter SLC14A1. Cohort studies have shown that SLC14A1-positive CAF correlates with poor chemotherapy response and prognosis in clinical patients. Mechanistically, SLC14A1-positive CAFs promoted tumor stemness via the WNT5A/β-catenin pathway, and the cGAS-STING pathway maintained this particular phenotype. The tumor-promoting effect of this CAF was remarkably weakened after the use of an inhibitor of STING or cGAS to suppress this pathway [454]. Combined therapy has been described in bladder cancer as well. The efficacy of dual suppression of TGF-β and PD-1 pathway utilizing Vactosertib (TEW-7197) and Durvalumab has been investigated in an open-labeled phase II study (NCT04064190). The study is not recruiting yet, and it is anticipated that a total of 48 patients will be enrolled to evaluate whether the cooperation of Vactosertib and Durvalumab can increase patients’ ORR. Durvalumab will be administered with the standard regimen of 1500 mg intravenously every four weeks. Vactosertib will be administered at a dose of 300 mg orally twice a day for 5 days per week, and all treatments will be administered for up to two years. Notably, the aforementioned exosome miR-146a-5p has recently been revealed to have therapeutic potential in bladder cancer and may be highly correlated with urothelial bladder cancer recurrence, as upregulated miR-146a-5p can promote cancer stemness as well as chemotherapy resistance [455].

**Table 3:** Clinical trials targeting/relating to the CAF

**Target**	**Drug**	**Combination** **agent**	**Phase**	**Tumor type**	**Clinical efficacy**	**PFS(m)**	**OS(m)**	**Safety**	**NCT Number**	**Status**
FAP	68Ga-FAP-2286	-	Phase 1	Metastatic solid cancer	-	-	-	-	NCT04621435	Recruiting
	68Ga-FAPI-04	-	Phase 2	Epithelial ovarian cancer	-	-	-	-	NCT04504110	Unknown
		-	Phase 1	Various cancer types	-	-	-	-	NCT04459273	Recruiting
	[18F] FAPI-74 PET/CT	-	Phase 2	Gastrointestinal cancer	-	-	-	-	NCT05641896	Recruiting
	RO6874281	Trastuzumab or cetuximab	Phase 1	BC and HNC	-	-	-	-	NCT02627274	Completed
	AVA6000	-	Phase 1	Various solid tumors	-	-	-	-	NCT04969835	Recruiting
	Re-directed T cells	-	Phase 1	MPM	-	-	-	-	NCT01722149	Completed
	BXCL701	Pembrolizumab	Phase 1Phase 2	Prostate cancer	-	-	-	-	NCT03910660	Active, not recruiting
		Pembrolizumab	Phase 2	Metastatic PDAC	-	-	-	-	NCT05558982	Not yet recruiting
CXCR4	AMD3100	Cemiplimab	Phase 2	Metastatic PAC	-	-	-	-	NCT04177810	Completed
	BMS-936564	Nivolumab	Phase 1 Phase 2	PAC and SCLC	-	-	-	Serious adverse events: 31/41 Immune-mediated adverse events:4/41	NCT02472977	Terminated
	BL-8040	Pembrolizumab	Phase 2	Metastatic PAC	In cohort 1, the DCR was 34.5% in the evaluable population (modified intention to treat, mITT; N = 29), including nine patients (31%) with stable disease and one patient (3.4%) with partial response. In cohort 2, 22 patients received BL-8040 and pembrolizumab with chemotherapy, with an ORR, DCR, and median duration of response of 32%, 77%, and 7.8 months, respectively.	-	3.3 months in cohort 1	A total of 37 patients were enrolled in cohort 1, the most common adverse event was mild to moderate injection site reaction. Only one patient (2.7%) had permanent discontinuation of study drugs owing to treatment-related adverse events (pain and pruritus at the injection site), and no treatment-related deaths were observed	NCT02826486	Completed
		Cemiplimab, gemcitabine and nab-paclitaxel	Phase 2	Pancreas adenocarcinoma	-	-	-	-	NCT04543071	Recruiting
	MSX-122	-	Phase 1	Refractory metastatic or locally advanced solid tumors	-	-	-	-	NCT00591682	Suspended
	POL6326	Eribulin	Phase 1	Metastatic BC	Objective responses (all partial responses) were observed in 16 (30%; 95% CI 18–44) of 54 patients who were evaluable for antitumour activity.	4.6 months (95%CI 3.1–5.7) in the overall efficacy population.	-	The most common treatment-emergent adverse events of any grade were fatigue (44 [79%] of 56 patients), neutropenia (32 [57%]), infusion-related reactions (27 [48%]), alopecia (26 [46%]), constipation (26 [46%]), and nausea (25 [45%]). Serious adverse events occurred in 21 (38%) of 56 patients, including febrile neutropenia in five (9%) of 56 patients, neutrophil count decrease in two (4%) patients, constipation in two (4%) patients, pneumonia in two (4%) patients, and urinary tract infection in three (5%) patients. Two (4%) of 56 patients died while receiving study treatment; one from septic shock and one from pneumonia.	NCT01837095	Completed
TGF-β and PD-L1	M7824	-	Phase 1	Stage II-III HER2 positive breast cancer	-	-	-	-	NCT03620201	Active, not recruiting
	SAR439459	Cemiplimab	Phase 1	Malignant solid tumor	-	-	-	-	NCT03192345	Terminated
		Cemiplimab	Phase 1	Advanced or unresectable solid tumor	-	-	-	-	NCT04729725	Terminated
	TEW-7197	Durvalumab	Phase 2	Urothelial cancer	-	-	-	-	NCT04064190	Not yet recruiting
	LY2157299	Nivolumab	Phase 1 Phase 2	Advanced refractory solid tumors, recurrent or refractory NSCLC, or HCC	In phase 2, researchers provide the date on ORR, ORR of galunisertib + nivolumab (NSCLC) group and galunisertib + nivolumab (HCC) group was 24% and 0%	In phase 2, researchers provide the date on mPFS, mPFS of galunisertib + nivolumab (NSCLC) group and galunisertib + nivolumab (HCC) group was 5.62 months and 5.39 months	In phase 2, researchers provide the date on mOS, mOS of galunisertib + nivolumab (NSCLC) group and galunisertib + nivolumab (HCC) group was 11.99 months and 14.52 months	Serious adverse events: 19/41	NCT02423343	Completed
TGF-β	Galunisertib	Paclitaxel	Phase 1	Metastatic androgen receptor negative (AR-) triple negative BC	-	-	-	-	NCT02672475	Active, not recruiting
	Fresolimumab	-	Phase 2	Metastatic BC	Abscopal response rate 100% for group: Fresolimumab 1 mg/kg and group; Fresolimumab 10 mg/kg	-	Arm 1: 7.57 months; arm 2: 16.0 months	Serious adverse events 3/11 for group: Fresolimumab 1 mg/kg and 3/12 for Fresolimumab 10 mg/kg	NCT01401062	Completed
	LY3200882	Capecitabine	Phase 1 Phase 2	Advanced resistant TGF-beta activated CRC	-	-	-	-	NCT04031872	Unknown
HSP90	XL888	Pembrolizumab	Phase 1	Advanced gastrointestinal cancer	-	-	-	-	NCT03095781	Active, not recruiting
Hedgehog	Sonidegib	Gemcitabine and nab paclitaxel	Phase 1 Phase 2	Pancreatic cancer	PR 13%, SD 58%, and PD 29%	-	6	six therapy-related grade 4 AEs and three grade 5 were observed	NCT02358161	Completed
Vitamin A	ATRA	Gemcitabine and nab-paclitaxel	Phase 2	Locally advanced PDAC	-	-	-	-	NCT04241276	Recruiting
HDAC, PI3K/AKT	CUDC-907	-	Phase 1	Advanced/relapsed solid tumors	-	-	-	-	NCT02307240	Completed
STAT3	BBI608	Paclitaxel	Phase 3	Non-squamous NSCLC	-	-	-	-	NCT02826161	Terminated
		Gemcitabine and nab-paclitaxel	Phase 3	Metastatic PDAC	DCR among napabucasin-treated and control-treated patients was 74.5% and 76.0%, respectively, and ORR was 43.2% and 42.9%, respectively	Napabucasin-treated group: 6.7 months, control-treated group: 6.1 months	Napabucasin-treated group: 11.4 months, control-treated group: 11.7 months	The most common AEs among napabucasin-treated and control-treated patients were diarrhoea (73.1% vs 38.9%), nausea (58.6% vs 46.1%), and anaemia (54.5% vs 58.1%). Serious AEs were reported in 58.8% of patients treated with napabucasin plus nab-paclitaxel with gemcitabine and 49.9% of those administered nab-paclitaxel with gemcitabine alone	NCT02993731	Completed
	BBI503 or BBI608	Sorafenib	Phase 1 Phase 2	HCC	Researchers provided data on ORR and DCR in phase 2 study. ORR based on RECIST 1.1 criteria among three arms was 3.6% (95%CI 0.1%-18.3%), 0 (95%CI 0-30.8%), 9.7% (95%CI 2%-25.8%), respectively. DCR based on RECIST 1.1 criteria among three arms was 35.7% (95%CI 18.6%-55.9%), 70% (95%CI 34.8%-93.3%), 48.4% (95%CI 30.2%-66.9%), respectively.	-	-	Serious adverse events: 39/91	NCT02279719	Completed
		Paclitaxel	Phase 1Phase 2	Advanced malignancies	Researchers provided data on ORR and DCR. ORR among five arms was 0%, 28.4%, 0%, 9.2% and 20.7%, respectively; DCR among five arms was 100%, 53.1%, 0%, 56.1% and 51.7%, respectively	Researchers provided data on mPFS, mPFS among five arms was NA, 2.23 months, NA, 2.07 months and 2.3 months, respectively	Researchers provided data on mOS, mOS among five arms was NA, 7.79 months, NA, 2.07 months and 2.3 months, respectively	Serious adverse events: 53/565	NCT01325441	Completed
LRRC15	ABBV-085	-	Phase1	Advanced solid tumors	In the “all sarcomas at all doses” population the ORR was 10.8%. In the patients with osteosarcoma or UPS treated at the 3.6 mg/kg dose the ORR was 20%. Among patients with UPS treated at 3.6 mg/kg, four patients had PR with tumor shrinkage of >30%	-	-	Most common treatment-related adverse events were fatigue, nausea, and decreased appetite	NCT02565758	Completed
Hyaluronic acid	PEGPH20	Avelumab	Early Phase 1	Chemotherapy resistant PAC	-	-	-	-	NCT03481920	Terminated
BET	GSK525762	-	Phase 1	NUT midline carcinoma and other cancers	Among 19 patients with NC, four achieved either confirmed or unconfirmed partial response, eight had stable disease as best response, and four were progression-free for more than 6 months.	Median PFS for the NC cohort was 2.5 months (95% confidence interval = 0.5 to 3.7 months).	-	The most frequent treatment-related adverse events of any grade were thrombocytopenia (51%) and gastrointestinal events, including nausea, vomiting, diarrhea, decreased appetite, and dysgeusia (22%–42%), anemia (22%), and fatigue (20%).	NCT01587703	Completed
ErbB3	MM-121	-	Phase 1	Advanced solid tumors	ORR was 0%	Median PFS estimate was 7.1 (95% CI: 4.7‒7.4) weeks in the dose escalation and 7.1 (95% CI: 6.6‒15.9) weeks in the dose expansion portion of the study	-	The most common TEAEs related to seribantumab were nausea (44%), diarrhea (36%), fatigue (28%), and skin rash (24%). No infusion-related reactions or dose-dependency were observed. Serious adverse events (SAEs) occurred in 24% (6/25) of patients in the dose escalation portion	NCT00734305	Completed
HER3	Seribantumab	-	Phase 2	NRG1 gene fusion positive advanced solid tumors	-	-	-	-	NCT04383210	Active, not recruiting
RTKs	Regorafenib	-	Phase 2	Gastric or gastroesophagel Junction Cancer	-	-	-	-	NCT03627728	Recruiting
		Nivolumab	Phase 3	Gastro-oesophageal cancer	-	-	-	-	NCT04879368	Recruiting
-	Gemcitabine	PEGPH20 and placebo	Phase 1 Phase 2	Stage IV Pancreatic Cancer	PEGPH20 1.0 μg/kg group: ORR=0% and DCR=25% PEGPH20 1.6 μg/kg group: ORR=50% and DCR=100% PEGPH20 3.0 μg/kg group: ORR=40% and DCR=70%	47 days for group: PEGPH20 1.0 μg/kg 276 days for group: PEGPH20 1.6 μg/kg 113 days for group: PEGPH20 3.0 μg/kg.	109.5 days for group: PEGPH20 1.0 μg/kg 199.5 days for group: PEGPH20 1.6 μg/kg 220 days for group: PEGPH20 3.0 μg/kg	Serious adverse events were 2/4 for group: PEGPH20 1.0 μg/kg, 1/4 for group: PEGPH20 1.6 μg/kg, and 13/20 for group: PEGPH20 3.0 μg/kg.	NCT01453153	Completed

*Abbreviations*: *BC* Breast cancer, *BCNS* Basal cell nevus syndrome, *CRC* Colorectal cancer, *DCR* Disease control rate, *HCC* Hepatocellular carcinoma, *HNC* Head and neck cancer, *laBCC* Locally advanced basal cell carcinoma, *mBCC* Metastatic basal cell carcinoma, *MPM* Malignant pleural mesothelioma, *NC* NUT midline carcinoma NSCLC, non-small Cell Lung Cancer, *ORR* Objective response rate, *OS* Overall survival, *PAC* Pancreatic cancer, *PDAC* Pancreatic ductal adenocarcinoma, *PFS* Progression-free survival, *PM* Peritoneal metastases, *SCLC* Small cell lung cancer

Due to the lack of fibroblasts in the central nervous system, it has been suggested that there is no CAF in glioblastoma. Therefore, research reports on glioma CAF are extremely rare. However, recent studies using spatial transcriptomics confirmed the existence of CAF in human glioblastoma and revealed the link between CAF and glioblastoma stem cell (GSC) [456]. Moreover, the TSP-4 secreted by CAF can improve the transcription level of HSF1 in glioblastoma cells and upregulate lncRNA DLEU1 to confer glioblastoma ferroptosis resistance [160]. Some of the previously mentioned agents are effective in animal glioma models. AMD3100 encapsulated in synthetic protein nanoparticles (AMD3100-SPNPs) has been validated to diminish glioma growth and restore radiation sensitivity in GBM mouse models in vivo [457]. Again, NT157 has been found to inhibit glioma cell proliferation and sensitize glioma cells to apoptosis induced by tumor necrosis factor-related apoptosis-induced ligand (TRAIL) via DR5 upregulation [458]. However, their effects on human glioma have yet to be demonstrated.

#### Progression in clinical trials of targeted CAF

Although animal experiments still dominate research on CAFs, some clinical trials have shown unsatisfactory results, which may be related to the complex heterogeneity of CAFs. There are still some clinical trials underway, as previously reported. We highlight recent advances in CAF-related clinical trials in multiple cancers in Table 3. These clinical trials include not only those targeting CAF-expressed markers but also those targeting CAF-related pathways. 

#### Conclusion and future perspectives

The relationship between tumors and TME is like fish and water, in which TME greatly facilitates the proliferation and progression of tumors. Since CAF is an integral part of TME, CAF-targeted therapy has significantly progressed in recent years. The advent of single-cell sequencing and spatial single-cell sequencing has made it possible to analyze specific CAF biomarkers and identify CAF subpopulations. These new technologies ensure the rapid exploration of CAF-specific markers and the development of CAF-specific diagnostic or prognostic protocols. Swift advances in CAF biology have laid a solid foundation for developing novel therapeutic strategies targeting these cells in cancer therapy. However, it is indisputable that CAF has exhibited the juxtaposition of tumor-promoting and tumor-repressing functions. The inherent phenotypic and functional heterogeneity of CAFs may arise from their different cellular origins and thus requires wary consideration when designing CAF-targeted tumor therapies. CAF-targeted therapeutic strategies targeting surface markers, relevant effector molecules, their associated signaling pathways and restricting ECM remodeling have been developed. The main objectives of these approaches are direct or tangential depletion of CAFs, reduction or elimination of their immunosuppressive and tumor-promoting properties, and normalization or reprogramming of CAFs to a quiescent state.

Currently, CAF-based therapeutic strategies are mainly developed in breast cancer, pancreatic cancer, lung cancer, colorectal cancer, prostate cancer, and melanoma. The efficacy of CAF-based therapeutic strategies is expected to be further validated in more cancers. With ongoing research into the molecular mechanisms underlying CAF pathology, many drugs targeting critical regulators of CAF are undergoing clinical and preclinical evaluation. However, it can be concluded that most are phase II clinical trials, and only a few entered phase III clinical trials. Besides, tumor clinical trials directly targeting CAFs still are urgently needed and should be improved.

Of note, targeting CAF strategies has faced several obstacles, including the shortage of CAF-specific biomarkers and their functional heterogeneity. Some studies have unraveled that inhibition of CAF function alone appears to directly inhibit tumor growth, possibly because inhibition of promotional CAF function outweighs inhibition of inhibitory CAF function, as there is also evidence that some CAF subtypes promote tumor development when inhibited. Given that apCAFs are involved in anti-tumor immunity, the mere elimination of CAF presumably triggers serious, unpredictable biological consequences and could end up in crisis. Moreover, an experiment performed in a PDAC mouse model showed that while depletion of αSMA + myofibroblasts reduced fibrosis, an increase in tumor invasion was observed, and gemcitabine did not improve efficacy as a result, which led to diminished animal survival [101]. Thus, due to technological advances such as single-cell sequencing and novel biomaterials for cell-type-specific delivery, selective elimination of tumor-promoting CAF subpopulations or reversal of their tumor-promoting activity may become a strategy that can be used alone or in combination with other oncology therapies.

Other strategies are proposed in this scenario except for accurately targeting CAF subsets. Targeting transcription through cis-regulatory elements (CREs), such as promoters, enhancers, etc., has been presented as a possible method to target CAF. The use of CAFs as an alternate vector for administering anti-fibroblastic tumor medications has been investigated. Additionally, recent research has shown that fibroblasts have drug-clearing mechanisms. Due to this mechanism, CAFs take up gemcitabine more rapidly than pancreatic tumor cells, implying that targeting metabolic processes rather than removing CAFs altogether may modify the matrix and improve the bioavailability of chemotherapeutic agents in tumors. More importantly, although CAFs are attractive targets in tumors, targeting CAFs may also elicit unpredictable multifaceted stromal responses in the TME that may be patient-heterogeneous due to the complex intercellular interactions involving CAFs in TME. For instance, CAF interacts with tumor-infiltrating immune cells, including TAM, mast cell, NK, and DC, and immune components, including chemokines, cytokines, and effector molecules, to form an immunosuppressive TME. It is expected that therapeutic strategies targeting the cellular interaction between CAF and tumor-infiltrating immune cells could be efficient as well.

Eventually, the aforementioned studies proved that different subtypes of CAF can be converted into each other. This concept was recently confirmed by scientists in PDAC. Synthetic retinol Am80 impeded tissue sclerosis by preventing fiber cross-linking, thereby improving drug delivery efficiency, which was pertaining to the increased expression of the inhibitory biomarker Meflin in CAF, making it more of a tumor suppressor than a tumor promotion [459]. Therefore, when the treatment for CAF is challenging to carry out, induction of CAF conversion may become an effective treatment modality rather than directly targeting CAF. The synergistic combination of CAF-targeted therapy and other effective treatments, such as chemotherapy, radiotherapy, and immune checkpoint therapy, should also be considered for ultimate tumor eradication.

## Data Availability

Not applicable.
